# Dual DNA replication modes: varying fork speeds and initiation rates within the spatial replication program in *Xenopus*

**DOI:** 10.1093/nar/gkaf007

**Published:** 2025-01-30

**Authors:** Diletta Ciardo, Olivier Haccard, Francesco de Carli, Olivier Hyrien, Arach Goldar, Kathrin Marheineke

**Affiliations:** Institut de Biologie de l’Ecole Normale Supérieure, Ecole Normale Supérieure, CNRS, INSERM, Université PSL, F-75005 Paris, France; Université Paris-Saclay, CNRS, Institut des Neurosciences Paris-Saclay (NeuroPsi), F-91400 Saclay, France; Institut de Biologie de l’Ecole Normale Supérieure, Ecole Normale Supérieure, CNRS, INSERM, Université PSL, F-75005 Paris, France; Institut de Biologie de l’Ecole Normale Supérieure, Ecole Normale Supérieure, CNRS, INSERM, Université PSL, F-75005 Paris, France; Université Paris-Saclay, CEA, CNRS, Institute for Integrative Biology of the Cell, F-91190 Gif-sur-Yvette, France; Université Paris Cité, CNRS, Institut Jacques Monod, F-75013 Paris, France

## Abstract

Large vertebrate genomes duplicate by activating tens of thousands of DNA replication origins, irregularly spaced along the genome. The spatial and temporal regulation of the replication process is not yet fully understood. To investigate the DNA replication dynamics, we developed a methodology called RepliCorr, which uses the spatial correlation between replication patterns observed on stretched single-molecule DNA obtained by either DNA combing or high-throughput optical mapping. The analysis revealed two independent spatiotemporal processes that regulate the replication dynamics in the *Xenopus* model system. These mechanisms are referred to as a fast and a slow replication mode, differing by their opposite replication fork speed and rate of origin firing. We found that Polo-like kinase 1 (Plk1) depletion abolished the spatial separation of these two replication modes. In contrast, neither replication checkpoint inhibition nor Rap1-interacting factor (Rif1) depletion affected the distribution of these replication patterns. These results suggest that Plk1 plays an essential role in the local coordination of the spatial replication program and the initiation–elongation coupling along the chromosomes in *Xenopus*, ensuring the timely completion of the S phase.

## Graphical Abstract

**Graphical Abstract:**
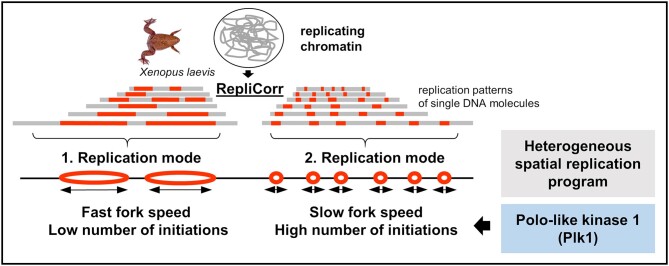

## Introduction

The faithful duplication of the genome is an essential and challenging event for all cells because it must robustly ensure efficient proliferation while maintaining genome stability. DNA replication starts from sites called replication origins; tens of thousands of origins are activated according to a regulated spatial and temporal program in each vertebrate cell to duplicate the chromosomes in a limited time window during the cell cycle. To better understand this process, the quantitative characterization of the highly heterogeneous replication dynamics constitutes an important step. Single DNA molecule data revealed that replication origins are spatially organized into clusters of 2–10 that fire nearly synchronously at different times during the S phase in mammalian cultured cells and in *Xenopus* (1–5). The *Xenopus in vitro* system recapitulates many aspects of cellular DNA replication. Replication-competent *Xenopus* egg extracts contain abundant maternal proteins and mimic the first rapid embryonic cell cycle when sperm nuclei are used as DNA templates (6). We and others have shown that in this system replication initiates at 5- to 15-kb intervals (3,4,7) and that the number of activated origins per time unit per length unit of unreplicated DNA, known as the initiation rate, increases to reach a maximum during the mid-late S phase before declining at the end of the S phase (8). This bell-shaped curve of the initiation rate was found to be universal for the DNA replication kinetics from yeast to humans despite differences in origin specification and S phase length (9,10). The replication kinetics are considered to emerge from stochastic initiation in all eukaryotes (11,12). In *Xenopus*, we recently showed by numerical simulations that the genome could be segmented into regions of high and low probabilities of origin firing (13), similar to early and late replication timing domains in other model systems. This and other common DNA replication models assume that the replication fork speed is constant (9,14). However, single-molecule methods revealed that the fork speed is heterogeneous in mammalian cells (1,15), *Xenopus* (5) and *Saccharomyces cerevisiae* (16). To ensure timely S phase completion, the fork speed and initiation rate are coordinated to compensate for varying replicon sizes or fork stalling. Several studies in mammalian cells, *Xenopus* and budding yeast have shown that artificial fork slowing or stalling leads to the activation of dormant origins (17–19). On the other side, decreasing initiation can lead to an increase in the fork speed (20–23). A similar fork speed and initiation rate coupling has been reported in early mouse embryos (24). Fork speed and inter-origin distances (IODs) are very low in two-cell embryos but increase progressively and coordinately during later developmental stages. However, the mechanism(s) underlying this correlation remains unclear. More than fifty protein factors spatially and temporally regulate the coordinated activation of replication origins. Two S-phase-specific kinases, cyclin-dependent kinases (CDK) and Dbf4-dependent kinases (DDK), are necessary for the activation of replication origins at the G1/S phase transition (25). They are counteracted by two independent pathways to negatively regulate the spatiotemporal program. The ATR/Chk1-dependent replication checkpoint pathway inhibits the activation of late replication origins in yeasts (26–28), *Xenopus* (5,29–31) and mammalian cells (32,33), by targeting CDK and DDK kinases. On the other hand, the replication timing regulator Rap1-interacting factor (Rif1) has been shown to inhibit late replication in yeast (34,35), mice (36) and human cell culture lines (37) at the level of large chromatin domains. In *Xenopus*, the depletion of Rif1 accelerates the replication program by accelerating origin cluster activation and increasing replication foci number (38). Rif1 opposes replicative helicase activation by recruiting the protein phosphatase 1 (PP1) close to origins, thereby counteracting DDK-mediated MCM2–7 activation (39–41). Finally, Polo-like kinase 1 (Plk1), mainly known to regulate mitosis, checkpoint recovery and adaptation (42), is also a positive regulator of the replication program. During the G1 phase, Plk1 promotes pre-replication complex loading or maintenance in mammalian cells, which leads to an increase in DNA synthesis. (43,44). It also promotes origin activation in the *Xenopus in vitro* system by inhibiting the replication checkpoint and Rif1 (45–47). However, the role of these three regulatory pathways in the spatial organization of the replication process is poorly understood.

To address this question, we developed a powerful and robust analysis approach named RepliCorr, which facilitates the quantitative characterization of replication patterns measured on stretched DNA molecules during DNA combing and high-throughput optical mapping experiments. RepliCorr revealed two spatially and temporally separated replication processes in the *Xenopus in vitro* system. The first process shows a fast replication fork speed coupled with a low initiation rate, whereas the second process shows a slow replication fork speed associated with a high initiation rate. We used RepliCorr to analyze experiments in which three regulatory pathways were disrupted (31,47,38). Chk1 inhibition or overexpression and Rif1 depletion did not affect the organization of these two processes. However, the depletion of Plk1 canceled out this dynamic separation. These results strongly suggest that Plk1 regulates the spatial replication program and the coupling between initiation and elongation in early *Xenopus* embryos to ensure the timely completion of the S phase.

## Materials and methods

### DNA combing experiments in the *Xenopus in vitro* system and data analysis

DNA combing data were chosen from experiments during a control S phase and after Plk1 depletion (47), Rif1 depletion (38) or after Chk1 inhibition by UCN-01 and Chk1 overexpression (31). Detailed experimental conditions and primary analysis are described in the respective publications. Briefly, sperm nuclei (2000 nuclei/µl) were replicated in egg extracts in the presence of biotin-dUTP naturally synchronously; genomic DNA was isolated at different times during the S phase and stretched on silanized coverslips. After immunolabeling, images were captured using a fluorescence microscope, and replication eyes were defined as the incorporation tracks of biotin-dUTP on DNA molecules. Each molecule was measured using Fiji software and compiled using macros in Microsoft Excel. The replicated fraction *f* of each fiber was calculated as the sum of eye lengths (red tracks, Streptavidin Alexa Fluor 594) divided by the total DNA length (green track, anti-DNA antibody, Alexa Fluor 488). The initiation rate was calculated as follows:


(1) \begin{eqnarray*} I(f)=\frac{N}{L(1-f)\Delta t}, \end{eqnarray*}

where *N* represents the number of new initiations defined as replication eyes <3 kb, *L* is the length of the fiber, *f* is the replicated fraction of the DNA molecule, and Δ*t* = 180 s is the time interval in which a detectable initiation event can occur, considering that the average replication fork speed in the *Xenopus in vitro* system is ∼0.5 kb/min (5). After identifying replicated and unreplicated tracks on each DNA molecule, we constructed a binary signal where ‘1’ and ‘0’ were assigned to replicated and unreplicated units, respectively. To obtain the auto-correlation function of the fluorescence intensity profile of each molecule, we used the unbiased estimate of the cross-correlation (*xcorr*) function in Matlab (vR2013a):


(2) \begin{eqnarray*} C(r)=\left \lbrace \begin{array}{l{@}{\quad}l} \frac{1}{n-|r|}\sum \limits _{n=0}^{N-r-1} x_{n+r}x_n , & \text{if}\ r\ >\ 0,\\ C(-r), & \text{if}\ r\ <\ 0, \end{array}\right. \end{eqnarray*}

where *x*_*n*_ corresponds to the binarized signal at the position *n*. DNA molecules (>80 kb) were selected and ordered by the replicated fraction and grouped in bins of different sizes depending on the sample. Bins were *f*_1_ = (0, 0.11], *f*_2_ = (0.11, 0.21], *f*_3_ = (0.21, 0.32], *f*_4_ = (0.32, 0.42], *f*_5_ = (0.42, 0.54], *f*_6_ = (0.54, 0.64], and *f*_7_ = (0.64, 0.75]. Thus, fibers with a replication content 0% and >75% are excluded from the analysis. The averaged initiation rate and correlation function were calculated and plotted as a function of the averaged replicated fraction for the molecules in the bins.

### HOMARD data analysis

Images of replicating DNA molecules from sperm nuclei in egg extracts were obtained by High-throughput Optical Mapping of Replicating DNA (HOMARD) using the NanoChannel Array Irys^®^ System (Bionano Genomics) as described (48) using the same fluorescent labeling strategy as in OMAR (49). In total, 100 580 fibers from nuclei stopped in the early S phase (35 min), and 47 915 fibers from nuclei stopped in the late S phase (120 min). The fibers were visualized in blue for total DNA (Yoyo-1) and red for replicating tracks after directly incorporating AlexaFluor 647 aha-dUTP. Images were corrected for chromatic focal aberration to superimpose the blue and red channels exactly. The replicating signal detected along each stained DNA molecule was binarized using a standard thresholding method. The replication fraction *f* of each fiber was defined as the average of its binary signal. The correlation between fiber profiles was performed on the binary signal.

### Monte Carlo simulation of DNA replication process

A Monte Carlo method was used to simulate DNA replication, as previously defined in (8). In the simulation, 100 DNA molecules were reproduced as a 1D array of 150 blocks with a value 1 for replicated DNA and 0 for unreplicated DNA. Each block was considered as 1 kb. At each step, the origins to activate were selected depending on the probability of initiation *P*(*t*). Initiation was allowed only in the unreplicated fraction of the simulated fibers. At each step, to reproduce DNA elongation, forks move by one block. For each simulation, a constant speed was fixed as $v$ = *N*kb/min. Then, the interval between two consecutive steps of the simulation was defined by the time necessary to replicate one block by a single fork and was set equal to 1/*v*. As in the KJMA models, the critical nucleus size (above which nuclei grow but below which they dissolve) is considered infinitesimal, the activation of an origin at a given position does not induce the conversion of the block value from 0 to 1. The initiation will be visible only at the following step due to the elongation.

### Model of the auto-correlation function of fluorescence profiles of replicated DNA molecules

We considered that the dynamics of DNA replication along the genome are analogous to the 1D nucleation and growth process, as previously described (50–52). The rate of origin firing per time unit and length of unreplicated DNA is temporally scale-free (52). We then assumed this explicit form for the initiation frequency as a function of time: *I*(*t*) = *I*_0_*t*^α^, with *I*_0_ ≥ 0 and α ≥ 0. This expression is a good approximation for the increasing region of the initiation frequency, to which we restricted the analysis. The parameter α summarizes how fast the frequency of initiation increases over time. By considering the work of Sekimoto (53), the replicated fraction of a molecule as a function of time *t* was expressed as


(3) \begin{eqnarray*} f(t)&=& 1-exp\bigg (-2vI_0\int _{0}^{t}{\rm d}t^{\prime }(t^{\prime })^\alpha (t-t^{\prime }) \bigg ) \nonumber\\ &=& 1-exp\bigg (-\frac{2vI_0t^{\alpha +2}}{(\alpha +1)(\alpha +2)} \bigg ). \end{eqnarray*}

The auto-correlation function of two points separated by a distance *r* at a certain time *t* was expressed as


(4) \begin{eqnarray*} C(r,t)&=& 1-2\phi (t)+\phi (t)^2 exp\bigg (I_0\int _{0}^{t-r/2v}{\rm d}t^{\prime }(t^{\prime })^\alpha [2v(t-t^{\prime })-r] \bigg ) \nonumber\\ &=& 1-2\phi (t)+\phi (t)^2 exp\bigg ( \frac{2vI_0t^{\alpha +2}}{(\alpha +1)(\alpha +2)}\bigg (1-\frac{r}{2vt} \bigg )^{\alpha +2} \bigg ), \end{eqnarray*}

where $v$ is replication fork speed and ϕ(*t*) = 1 − *f*(*t*) is the unreplicated fraction of a fiber. Equation (4) is valid for *r* < *l*_max_ = 2*vt*, where *l*_max_ represents the maximum replication eye length present at time *t*. Calculations are detailed in Supplementary methods.

### Parameters optimization

The replication parameters $v$, *I*_0_ and α of the model were estimated given the experimental frequency of initiation *I*(*f*) as a function of the replicated fraction *f* and the correlation function *C*(*r*, *f*) for different replicated fractions *f* as a function of *r*. The time was obtained from the analytical inversion of the Equation (3) as *t* = *f*^−1^($v$, *I*_0_, α). We used the genetic optimization algorithm on the Matlab platform (vR2012a) for parameter optimization. The fitness function was defined as the reduced χ^2^. In the genetic algorithm, we used 10 subpopulations of 10 individuals with a migration fraction of 0.1 and a migration interval of 50 steps. Each individual defined a set of variables for the fit, and the subpopulation variables were chosen within the bounds reported in Table 1. At each generation, one elite child was selected for the next generation. The rest of the population comprised 60% of children obtained after a scattered crossover between two individuals chosen by roulette wheel selection and 40% of children obtained by uniform mutation. The genetic algorithm was stopped after 3000 generations or if the fitness function attained a value of 0.5.

**Table 1.** Lower and upper bounds of the fit variables

Variable	Lower bound	Upper bound
$v$	1e−10	10
*I*_0_	1e−15	1
α	0	5

### Principal component analysis and agglomerative hierarchical clustering

DNA molecules >80 kb in length were ordered by the replicated fraction and grouped in bins of different sizes depending on the sample. A matrix was obtained for each replicated fraction bin: each row represented the correlation function of one molecule in the bin; each column represented the value of all the correlation functions for a specific distance *r*. We considered *r* in the interval of 0–25 kb. To reduce the dimensionality of the dataset, we performed principal component analysis (PCA) on the matrix of the correlation functions with the *princomp* function in Matlab. We then used agglomerative hierarchical clustering to group the correlation functions according to different numbers of clusters and the silhouette values as clustering evaluation criteria. More precisely, the pairwise distance between pairs of correlation functions in a given bin was calculated as one minus the correlation coefficient between the two curves (*pdist* function with *correlation* distance matrix) to obtain a ‘distance vector’. An agglomerative hierarchical cluster tree was created using a weighted average distance (WPGMA) linkage method (*linkage* function). Finally, we used the *cluster* function to cut the hierarchical tree into two to five clusters. For each configuration, the clustering solution was evaluated by calculating the average silhouette values of each data point. The silhouette value for each point was obtained as follows:


(5) \begin{eqnarray*} S_i = \frac{(b_i-a_i)}{max(a_i,b_i)}, \end{eqnarray*}

where *a*_*i*_ is the average distance from the *i*th point to the other points in the same cluster, and *b*_*i*_ is the minimum average distance from the *i*th point to points in a different cluster, minimized over clusters. The distance was calculated as one minus the correlation coefficient between points. The silhouette value for a point in a cluster measures how it is similar to other points in its cluster compared with points in different clusters and ranges from −1 to +1.

## Results

### Application of the auto-correlation function to replication patterns of single DNA molecules

**Figure 1.**
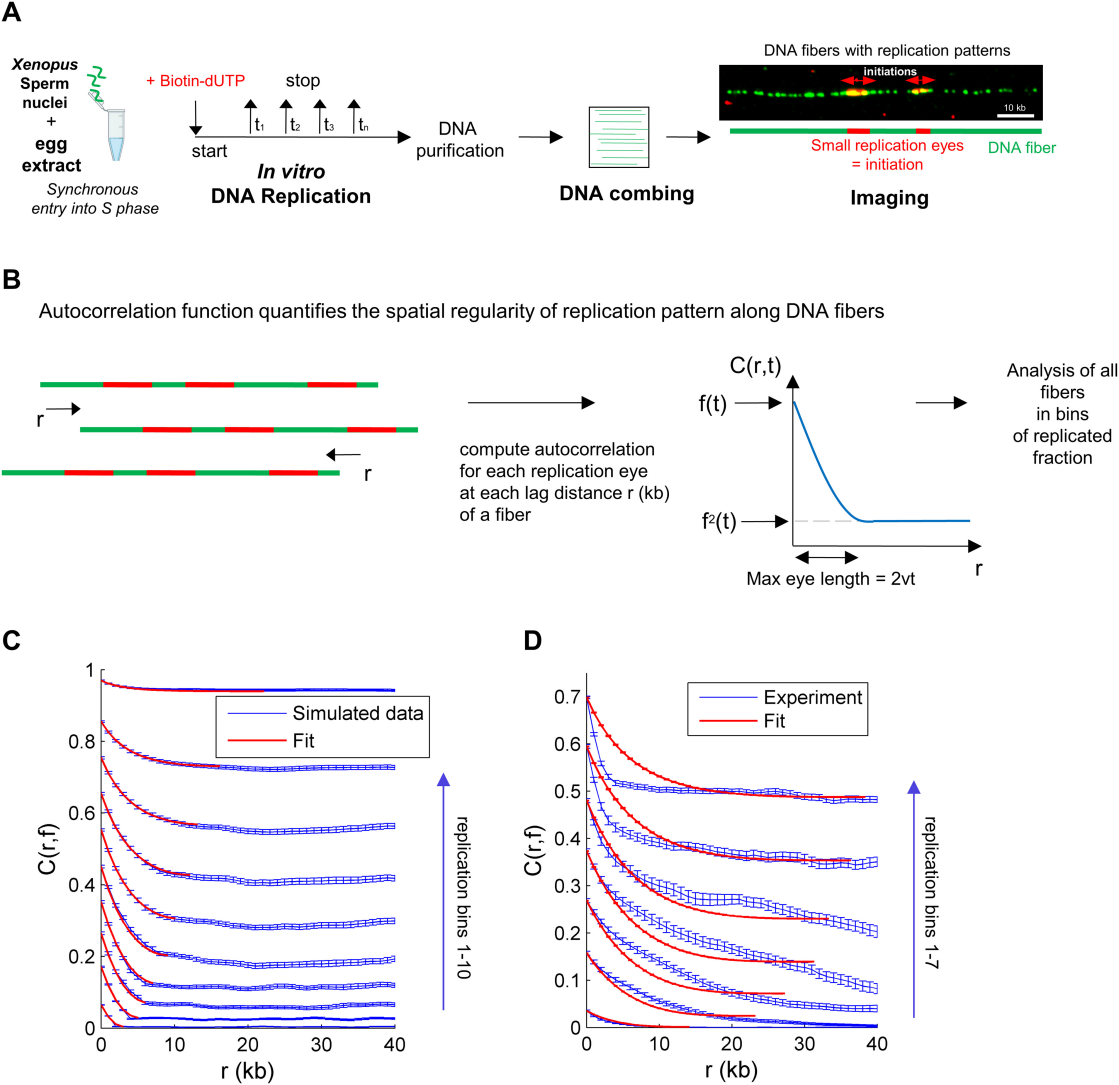
Filtering of replication patterns of single DNA molecules from DNA combing experiments during unperturbed S phase by an auto-correlation function *C*(*r*, *f*). (**A**) Workflow of DNA combing experiments in *the Xenopus in vitro* system. Sperm nuclei were incubated in egg extract in the presence of biotin-dUTP; replication reactions were stopped at different times during the unperturbed, naturally synchronous S phase; and DNA was purified and stretched onto coverslips. Replicated tracks on single DNA fibers were revealed by fluorescence microscopy after immunolabeling [replication eyes (red) and DNA molecule (green)]. (**B**) Auto-correlation function *C*(*r*) measures the spatial regularity of replication patterns of a DNA molecule and its shifted copies as a function of the lag distance *r*. (**C**) *C*(*r*, *f*) profile for a simulated dataset (mean with error, blue) for constant *I*(*f*) (0.03 kb^−1^ min^−1^) and constant $v$ (1 kb/min) and fit (red) with one process at different bins of replicated fractions *f*. (**D**) Mean simulated *C*(*r*, *f*) profiles with standard deviation for three independent control DNA combing experiment at different bins of replicated fractions *f* and fit with one process using the KJMA model. Bins of replicated fraction are *f*_1_ = (0, 0.11], *f*_2_ = (0.11, 0.21], *f*_3_ = (0.21, 0.32], *f*_4_ = (0.32, 0.42], *f*_5_ = (0.42, 0.54], *f*_6_ = (0.54, 0.64], and *f*_7_ = (0.64, 0.75].

To investigate the replication dynamics at the single-molecule level, we used DNA combing data for the unperturbed S phase in the *Xenopus* *in vitro system* (Figure 1A) (47). Next, the characteristics of observed replication patterns were obtained by filtering each DNA molecule using an auto-correlation function (Figure 1B), as described in the ‘Materials and methods’ section. Auto-correlation filtering quantifies the spatial regularity of the observed pattern. This widely used filtering has been successfully applied, e.g. to analyze the nucleosome positioning patterns (54). Here, we developed a new method to analyze the fluorescent signals from combed DNA molecules based on the KJMA model, which is detailed in the ‘Materials and methods’ section and Supplementary methods. We obtained an analytical expression for the auto-correlation (Equation 4). Applying this method to analyze the replication pattern of a single DNA molecule allowed us to extract the regularity of the replicated tracks. First, to check the general feasibility of this approach, we applied the auto-correlation function to a simulated dataset of replicated DNA molecules, as detailed in ‘Materials and methods’ section. In this dataset, we used constant values for initiation rate and fork speed and sorted the simulated replicated DNA fibers for their replication fraction into seven non-overlapping bins. We then calculated each molecule’s auto-correlation profile *C*(*r*, *f*) as a function of the lag distances *r* and their average replication fraction, *f* (Figure 1C, blue). A good fit of the simulated data was obtained with the analytical expression of Equation (4) (red), giving correct parameters (Supplementary Table S1). As expected by the formula, we found that at 0% replication, the *C*(*r*, *f*) was 0, whereas *C*(*r*, *f*) tended toward 1 as replication approached 100%. The maximal correlation value of each bin corresponds to the average replicated fraction of each bin. The slope for small lag distances *r* is given by the exponential decay constant 1/2*vt* in the expression of the correlation function (Equation 4). With increasing time or replicated fractions, the slope becomes shallower. Indeed, as the replication progresses, the average replicated track size increases. Hence, at short lag distances (*r* ≤ 2*vt*), *C*(*r*, *f*) highlights processes regulating DNA elongation. At long lag distances (*r* ≥ 2*vt*), *C*(*r*, *f*) reflects the distribution of activated replication origins on each DNA molecule.

We then applied this method to experimental data from three independent DNA combing experiments from the unperturbed S phase. We sorted the experimental replicated DNA molecules for their replicated fraction into seven non-overlapping bins. We then calculated for each DNA molecule the auto-correlation profile *C*(*r*, *f*) as a function of the lag distances *r* and their average replication fraction, *f*, (Figure 1D) and compared the experimental *C*(*r*, *f*) profiles to the simulated *C*(*r*, *f*) profiles (Figure 1C). As expected, as the replication degree of each bin increased, the slope of the averaged auto-correlation function *C*(*r*, *f*) became shallower for *r* ≤ 2*vt*. However, after the replication reached 40%, the slope of *C*(*r*, *f*) became sharper in the experimental profiles, whereas the slope in the simulated profiles became flatter. We interpret this transition as a change in the process regulating replication during the S phase. In addition, the fit of the experimental data with the correlation expression (red) did not reproduce the experimental profiles (blue), as it did in Figure 1C. Altogether, these results suggest that a single process may not regulate the replication process but that two or more independent processes may be necessary to explain the experimental profiles.

### The replication process results from a combination of two spatially separated fast and slow processes

**Figure 2.**
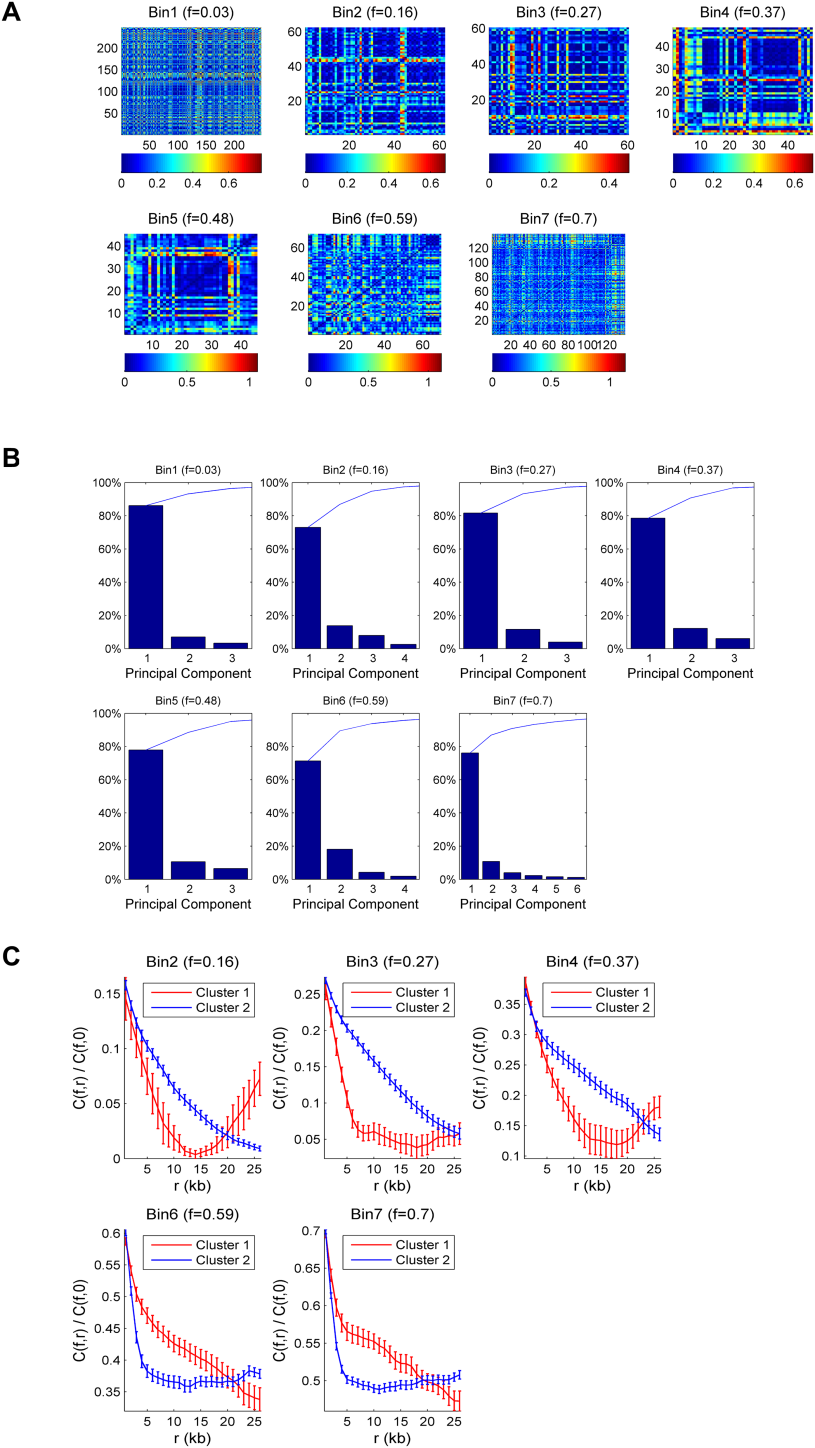
Hierarchical classification of DNA molecule’s replication patterns. (**A**) Similarity matrix between molecule’s *C*(*r*) for different replication fractions *f*. Each cell in the matrix represents the similarity *s* = 1 − pair-wise correlation coefficient between the *C*(*r*) of two molecules in the replication bin. A score *s* = 0 represents two perfectly similar *C*(*r*), and a score *s* = 1 represents two completely different *C*(*r*). (**B**) Pareto chart of the percent variability explained by each principal component. Each chart corresponds to a different interval of replicated fractions from 0 to 0.75; the average replicated fraction is reported on top. In each chart, the bars represent the percentage of variance described by the relative principal component in descending order. The line above represents the cumulative total. (**C**) Mean *C*(*r*, *f*) profiles for molecules hierarchically classified into two clusters, as suggested by the average silhouette value (Supplementary Figure S1). The *C*(*r*, *f*) of the category containing the smaller number of molecules is in cluster 1, and the *C*(*r*, *f*) of the category containing the larger number of molecules is in cluster 2. The fraction of molecules in each cluster is reported in Supplementary Figure S2. Error bars are standard deviations.

**Figure 3.**
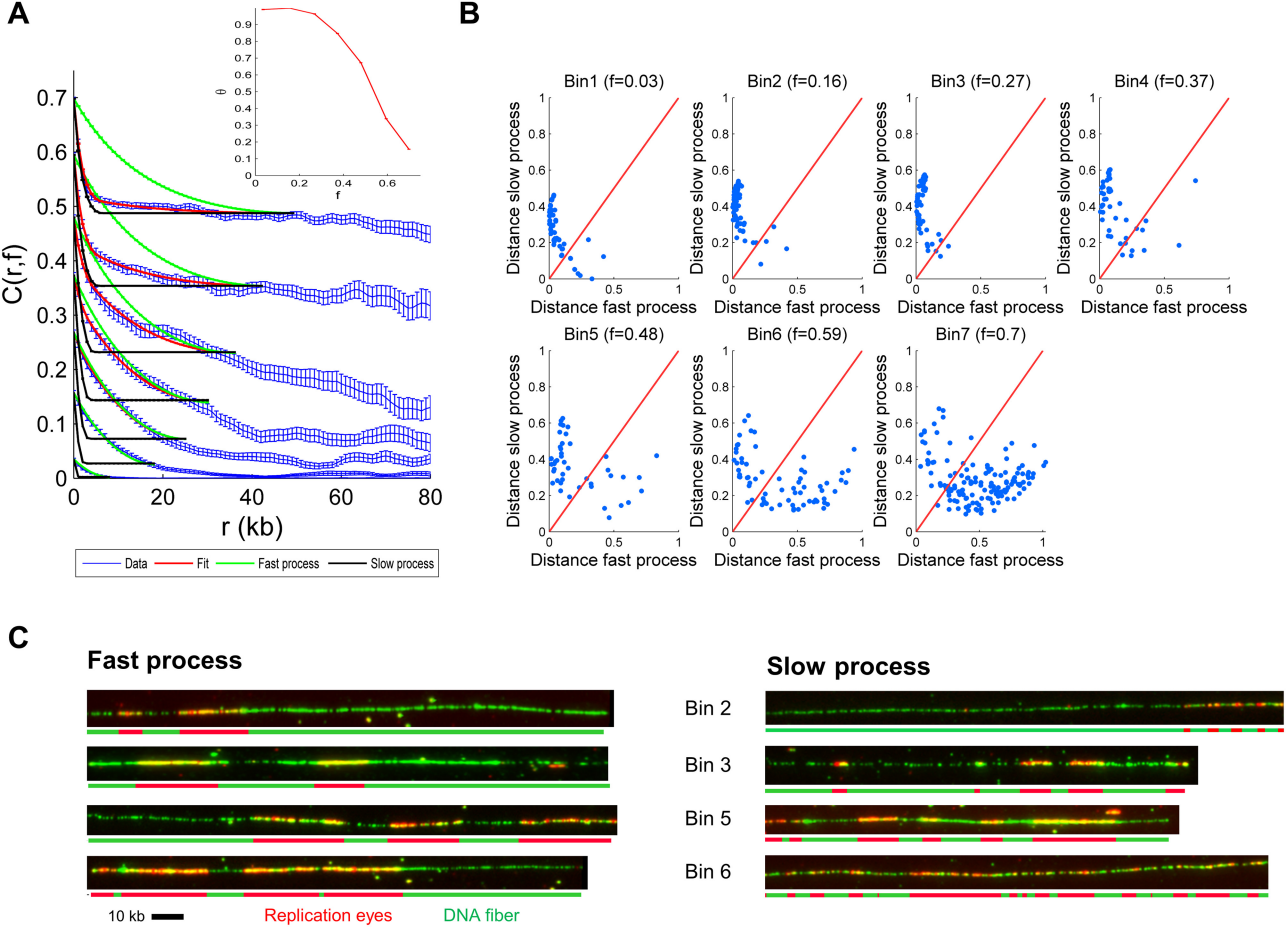
Categorization of DNA molecule’s replication patterns into two dynamical processes by RepliCorr. (**A**) Fit (red curve) of mean *C*(*r*, *f*) profile (blue curve, with standard deviation from three independent experiments) for each replication fraction bin calculated as *C*(*r*, *f*) = Θ(*f*)*C*1(*r*, *f*) + [1 − Θ(*f*)]*C2(*r*, *f*). The green curve is the correlation profile produced by the fast fork process [*C*1(*r*, *f*), model 1], and the black curve is the correlation profile produced by the slow fork process [*C*2(*r*, *f*), model 2]. The error bars are standard deviations. The inset is the Θ(*f*) profile. (**B**) Normalized correlation coefficients (ρ_1_, ρ_2_) between the molecule’s *C*(*r*, *f*) and *C*1(*r*, *f*) and *C*2(*r*, *f*) were calculated. The similarity distance between the molecule and each process was defined as 1 − ρ_1_ for the fast process 1 and 1 − ρ_2_ for the slow process and represented on a two orthogonal axis plot. The diagonal represents points of equal similarity to the two processes. Points above the diagonal are more similar to the fast process and points below are more similar to the slow process. (**C**) Representative fibers with replication patterns in the fast and slow process in the same replicated fraction bins. Bins of replicated fraction are *f*_1_ = (0, 0.11], *f*_2_ = (0.11, 0.21], *f*_3_ = (0.21, 0.32], *f*_4_ = (0.32, 0.42], *f*_5_ = (0.42, 0.54], *f*_6_ = (0.54, 0.64], and *f*_7_ = (0.64, 0.75].

To investigate this transition further, it is necessary to understand how to link the auto-correlation profiles of each fiber to the replication process that generates the observed replication patterns. To this aim, we assumed that our set of replicated molecules contains all the patterns the replication process can produce at a given replication fraction. To uncover potential similarities in the auto-correlation profiles, we constructed the correlation coefficient matrices for each replication bin, which allowed us to measure the degree of similarity between the auto-correlation profiles of different molecules (Figure 2A). In these matrices, each cell represents the similarity *s* = 1 − pair-wise correlation coefficient between the auto-correlation profile of one molecule and another molecule in the bin. A score close to 0 indicates perfect similarity between the two profiles, while a score close to 1 is the opposite. We observed considerable heterogeneity in the similarity scores, which confirms that the replication patterns are heterogeneous in each replication bin (47). Furthermore, a closer inspection of the similarity matrices shows a ‘plaid’ pattern, with many blocks consistently showing low similarity scores. This pattern corresponds to the presence of subgroups of DNA molecules that exhibit similar auto-correlation profiles and potentially similar replication behaviors. This observation suggests that molecules can be clustered into subgroups of similar auto-correlation profiles. To determine how molecules should be grouped, we reduced the dimension of the *s* matrix using a PCA. PCA revealed that only two independent linear combinations between scores were enough to describe >85% of the observed variability in measured *s* scores (Figure 2B, and Supplementary Figure S1). As the replication pattern of a molecule is the result of the stochastic activation of replication origins (11), using Kosambi–Karhunen–Loève theorem (55), we concluded that only two independent processes are enough to describe the diversity of observed auto-correlation profiles. Next, we compared the average *C*(*r*, *f*) of the two independent clusters at different replicated fractions, *f*. Interestingly, while for 0 ≤ *f* ≤ 0.5, molecules with a shallower slope (cluster 2) dominate in the population, an inversion occurred for 0.5 ≤ *f* ≤ 0.75 when molecules with a shallower slope (cluster 1) became less predominant (Figure 2C, and Supplementary Figure S2). We conclude that the replication dynamics can be represented as the superposition of two independent stochastic processes. Next, following the KJMA framework (51), we constructed a tractable mathematical model where the replication of a locus is induced by the simultaneous action of two independent processes (for more details, see Supplementary methods). Each process is characterized by the replication fork speed $v$ (kb/min) and the rate of replication origin activation *I*(*t*) = *I*_0_*t*^α^ (kb^−1^ min^−1^) per unit time per length of unreplicated DNA. Therefore, each process is defined by three parameters $v$, *I*_0_, α. Next, using this model, we expressed the auto-correlation profile *C*(*r*, *f*) of a molecule with a degree of replication *f* as *C*(*r*, *f*) = Θ(*f*)*C*_1_(*r*, *f*) + (1 − Θ(*f*))*C_2_(*r*, *f*), where Θ(*f*) is the mixing parameter between process 1 and 2 (0 ≤ Θ(*f*) ≤ 1). If they act alone, processes one and two create correlation profiles *C*_1_(*r*, *f*) and *C*_2_(*r*, *f*), respectively. After sorting fibers according to *f* and distributing them into seven bins, we modeled the averaged auto-correlation profile of each bin using the calculated *C*(*r*, *f*) (Figure 3A). For replicated fractions ≤0.4, the experimental correlation profiles were well reproduced by process 1 (green curve). In contrast, the average *C*(*r*, *f*) was predicted by process 2 (black curve) at higher replicated fractions. Fork velocity ($v$) and the initiation rate changed in opposite ways (Supplementary Table S2): process 1, named the ‘fast’ process, had a fast $v$ and a low initiation rate, and conversely, process 2, called ‘slow’, had a slow $v$ and high initiation rate. More precisely, the slow process presents a nearly 6-fold lower fork speed but an 8-fold higher initiation strength *I*_0_ than the fast process. To quantitatively model *C*(*r*, *f*), we introduced the mixing parameter Θ(*f*) (inset in Figure 3A) that acts as an external clock regulating the transition between the fast and slow process during the S phase. This suggests that an unknown replication-independent switch triggers the change of the replication dynamics in the cell. To further control for the necessity of two independent processes with different parameters, we also fitted the auto-correlation profiles and *I*(*f*) from the experimental data by considering two processes with either two different fork speeds and the same initiation rate or the same fork speed and two different initiation rates (Supplementary Figure S3A and B). In the first case, we could not fit very well the initiation rate and the time to replicate was too long to be compatible with the S phase length in this experimental system; in the second case, we could fit the initiation rate, but the time needed to replicate the fibers was again too high (Supplementary Table S2). Next, to investigate the distribution of DNA molecules between the two processes, we calculated the correlation coefficient, ρ, between the auto-correlation profile of each molecule and either *C*_1_(*r*, *f*) or *C*_2_(*r*, *f*) at a given replicated fraction. We defined the similarity distance as *s* = 1 − ρ. To visualize this distribution, we reported the similarity distance values for each fiber to the slow and fast process on a 2D graph where the *x*-axis represents the distance from the fast process, and the *y*-axis represents the distance from the slow process (Figure 3B). Since small *s* values indicate similarity to each process, points closer to the *y*-axis represent fibers from the fast process, and points closer to the *x*-axis represent points from the slow process. Interestingly, the data points are distributed vertically or horizontally, with few points around the diagonal. This suggests that the replication pattern of the majority of molecules is described exclusively either by the fast or the slow process alone. The fast process produces longer replicated tracks than the slow process for the same replicated fraction (Figure 3C, and Supplementary Figure S4). Thus, this single-molecule analysis method, which we call RepliCorr, unveils the spatial heterogeneity of fork speed and initiation rate along the genome. To investigate whether the same pattern distributions can also be observed at distinct times during the S phase, we analyzed the same dataset by individual timepoints taken throughout the S phase. Supplementary Figure S5 shows a very similar separation of distance patterns of the slow and fast process to Figure 3B, but with increasing S phase progression rather than increasing replication bins.

**Figure 4.**
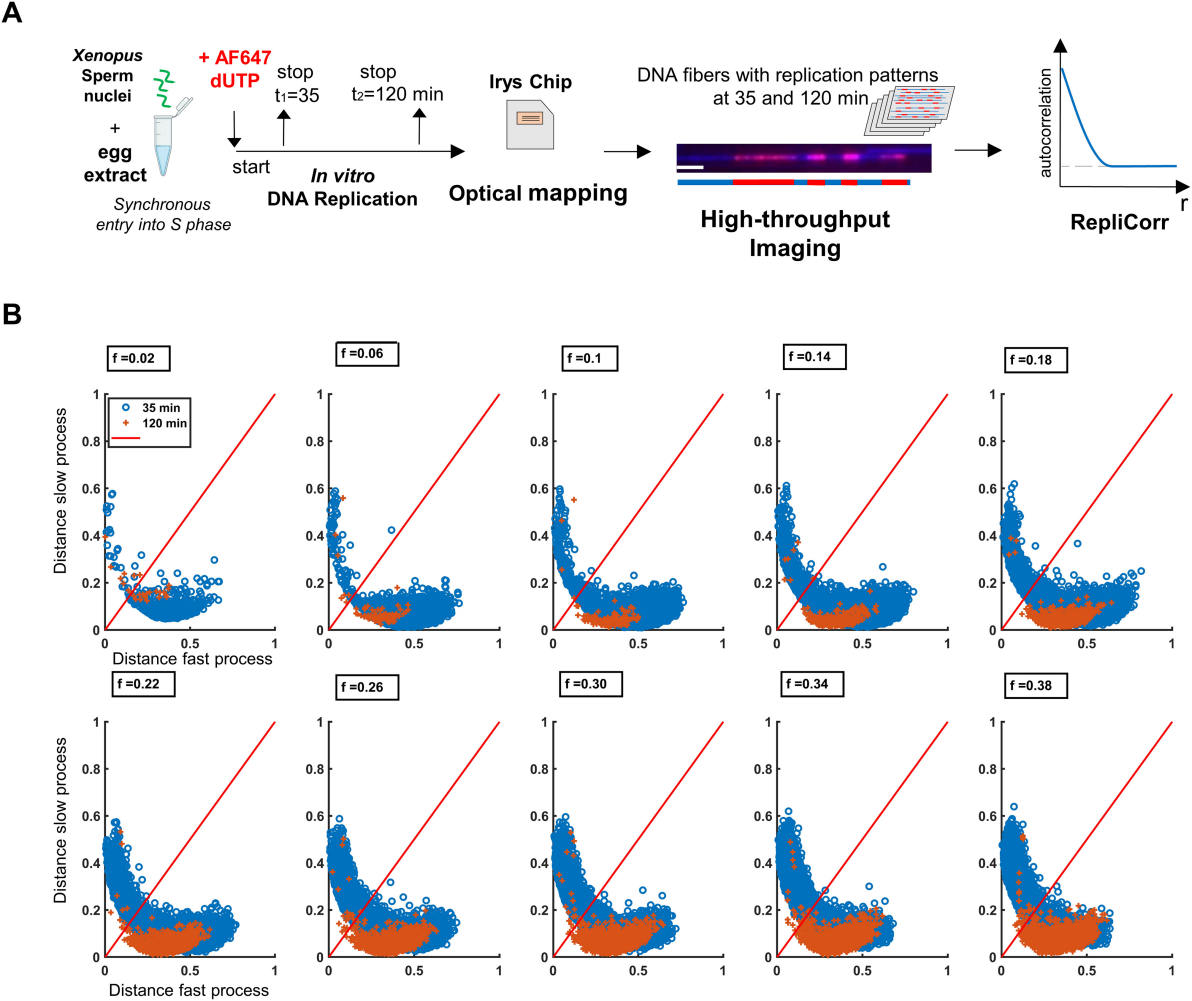
RepliCorr analysis of the HOMARD experiments in the *Xenopus in vitro* system. (**A**) Workflow of the experiment using HOMARD: Sperm nuclei were incubated in egg extracts in the presence of AF647-aha-dUTP, stopped in early (35 min) and late S phase (120 min), DNA was isolated and separated in Irys system with Yoyo-1 stain. Example DNA fiber with Bionano Irys system, Yoyo-1, whole DNA stain, small replication tracks (=initiations) labeled directly by AF647-aha-dUTP, early S phase (35 min), size bar 20 kb. (**B**) Normalized correlation coefficients (ρ_1_, ρ_2_) between the fibers’ *C*(*r,f*) and *C*1(*r*,*f*) and *C*2(r,*f*) were calculated. The similarity distance between the fiber and each process was defined as 1 − ρ_1_ for the fast process and 1 − ρ_2_ for the slow process and represented on a two-orthogonal axis plot. The diagonal represents points of equal similarity to the two processes;.

To verify whether the low-throughput of the DNA combing experiments (about 1000 fibers per condition and experiment) was sufficient for RepliCorr analysis, we analyzed unpublished high-throughput data (150 000 fibers), obtained by optical mapping of replicating *Xenopus* sperm DNA (HOMARD) in the *Xenopus in vitro* system (49) (Figure 4A, and Supplementary Figure S6). As for combed molecules, we modeled the auto-correlation profile of each molecule using *C*(*r*, *f*) = Θ(*f*)*C*_1_(*r*, *f*) + (1 − Θ(*f*))*C_2_(*r*, *f*). We calculated the distance between the experimentally defined auto-correlation profile of the fiber and the slow and fast process (Figure 4B). Due to the high-throughput of the optical mapping experiment, we could apply RepliCorr analysis to the early (35 min) and the late (120 min) timepoints, separately. As observed with the DNA combing, the data points were distributed vertically or horizontally, with few points around the diagonal direction for the early timepoint (blue points). However, late S phase patterns are distributed only along the axis of the slow process. This suggests that while both slow and fast processes coexisted in distinct genome regions in the early S phase, the slow process was nearly exclusive in the late S phase. Therefore, the low-throughput in DNA combing experiments influenced neither the outcome of the RepliCorr analysis nor the distribution of similarity distances along the graph representing the slow and fast processes. In addition, the high-throughput of the HOMARD analysis allowed us to visualize the temporal separation of these processes along the S phase. We conclude that in the *Xenopus in vitro* system DNA combing and HOMARD experiments show a clear spatial separation between the fast and the slow processes, as fibers are not distributed on the diagonal.

### Depletion of Plk1 reduces the spatial heterogeneity of the replication profiles

**Figure 5.**
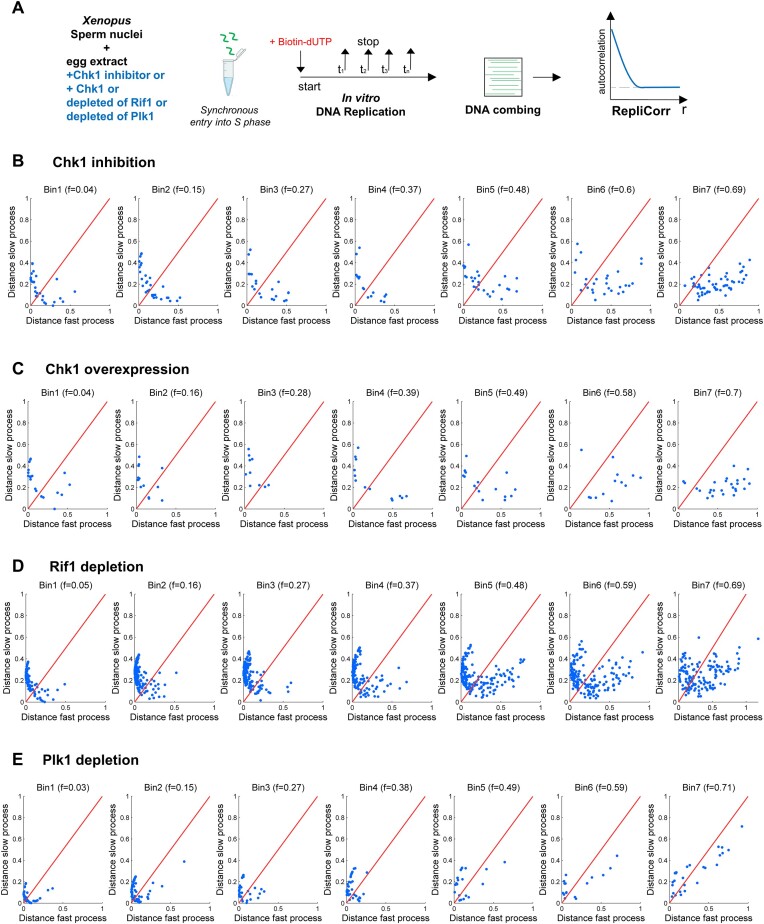
Strong modification of the replication patterns after Plk1 depletion but less after Chk1 inhibition, overexpression or Rif1 depletion. (**A**) An outline of experimental workflow: sperm nuclei were incubated in Chk1 inhibited or Chk1 overexpressed egg extracts or Rif1 or Plk1 immunodepleted egg extracts in the presence of biotin-dUTP. Genomic DNA was isolated at different times during the S phase, subjected to combing analysis and further analyzed by RepliCorr. Normalized correlation coefficients (ρ_1_, ρ_2_) between the fiber’s *C*(*r*, *f*) and *C*1(*r*, *f*) and *C*2(*r*, *f*) were calculated. The similarity distance between the fiber and each process was defined as 1 − ρ_1_ for the fast process and 1 − ρ_2_ for the slow process and represented on a two orthogonal axis plot. The diagonal represents points of equal similarity to the two processes. (**B**) Chk1 inhibition by UCN-01 (independent experiments *n* = 2). (**C**) Chk1 overexpression (*n* = 2). (**D**) Rif1 depletion (*n* = 2) and (**E**) Plk1 depletion (*n* = 3).

**Figure 6.**
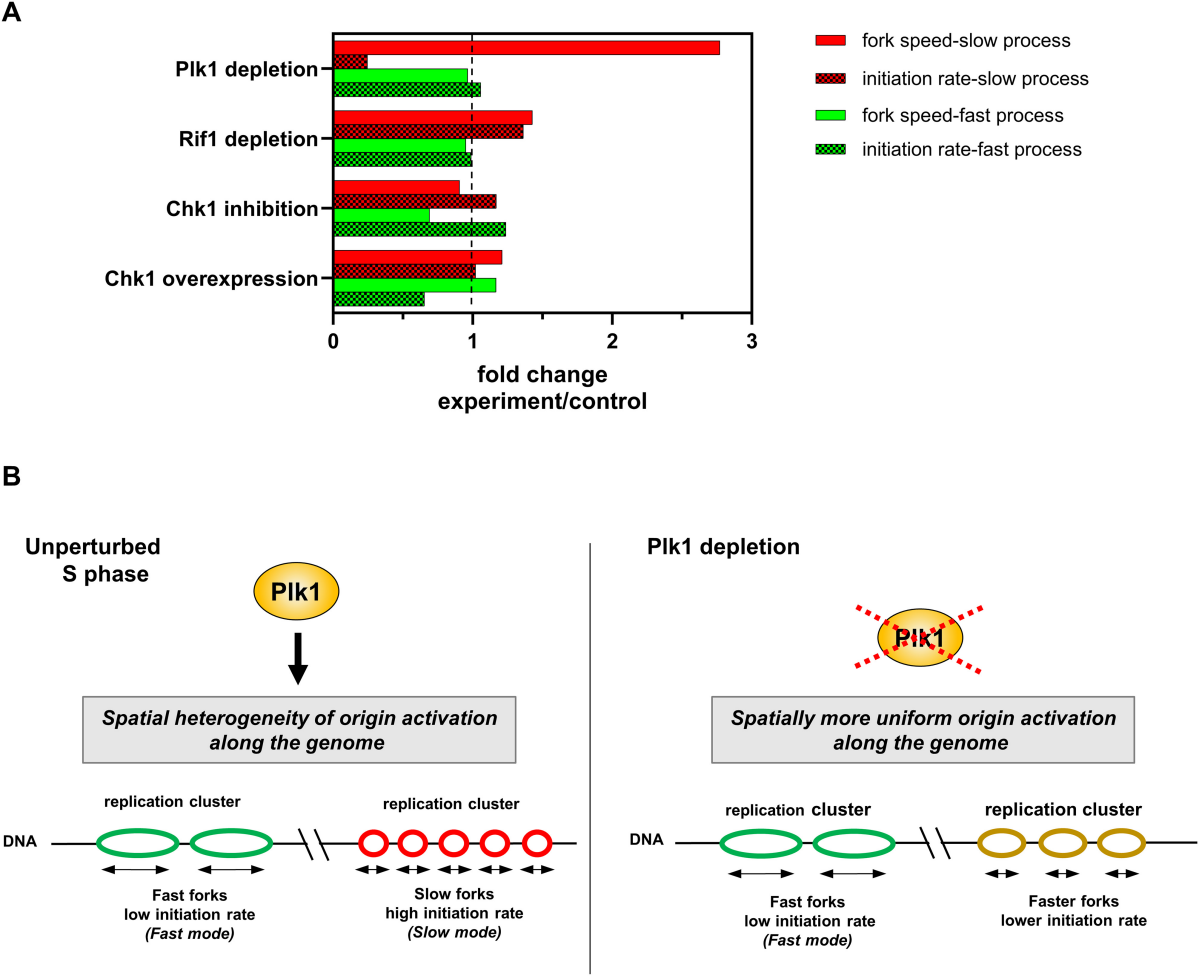
Plk1 depletion strongly increases fork speed and decreases initiation frequency in the slow process. (**A**) Comparison of initiation frequency and fork speed for the fast and slow replication process after Plk1 or Rif1 depletion and Chk1 inhibition or overexpression. Mean fold change of experiment/control for initiation frequencies per replicated fractions and fork speeds per experiment/control are shown for each replication mode, values from Supplementary Figure S10 and Supplementary Table S3, respectively. (**B**) Model of a Plk1-dependent regulation of the spatial replication program by a fast and slow replication process in *Xenopus*: in the presence of Plk1, two different replication patterns on DNA molecules can be distinguished, characterized by different fork speeds and initiation rates, leading to a non-uniform pattern of origin activation. Upon Plk1 depletion, origin activation along the genome becomes more homogeneous; the slow replication mode approaches the fast mode.

To identify molecular determinants involved in the spatial separation between the fast and slow process during the S phase, we used RepliCorr to analyze DNA combing data after inhibition or depletion of different known regulators of DNA replication (Figure 5A, and Supplementary Figure S7A–D). The ATR-Chk1 dependent intra-S phase checkpoint pathway inhibits origin firing at the level of replication clusters in *Xenopus* (5,31). Inhibition of the checkpoint effector kinase Chk1 by UCN-01 or Chk1 overexpression did not alter the partition of replicating DNA molecules into two separate classes (Figure 5B and C, and Supplementary Figure S8A and B). Another important negative regulator of the replication program in *Xenopus* is Rif1 (38). However, using RepliCorr, we found that after Rif1 depletion, the separation of molecules into the two classes was maintained (Figure 5D, and Supplementary Figure S8C). Still, slightly more data points were found around the diagonal, especially in higher replicated fraction bins compared with checkpoint-inhibited conditions. This suggests that the spatial heterogeneity of patterns is only slightly reduced after Rif1 depletion. We recently found by quantification of ${}^{32}{\mathbf {P}}$-dATP incorporation and DNA combing experiments that depletion of Plk1 inhibited DNA synthesis via inhibition of origin activation during normal S phase in *Xenopus*, whereas the add-back of recombinant Plk1 rescued DNA replication (46,47). Applying RepliCorr to replication patterns after Plk1 depletion showed a dramatic change in the replication pattern partition (Figure 5E), compared with the control (Figure 3B). The long and short replicated tracks were no longer spatially and temporarily separated but coexisted on molecules with a high degree of replication. A different representation of these distribution patterns further underlined the above observations (Supplementary Figure S9A and B). Interestingly, from the fits of the two independent processes of the correlation function and their individual parameter values (Supplementary Figure S10 and Supplementary Table S3), we noticed that after Plk1 depletion, the averaged initiation frequency decreased 4-fold and fork speed increased nearly 3-fold for the slow but not the fast process compared with the control (Figure 6A). Chk1 inhibition, overexpression or Rif1 depletion affected these processes much less than Plk1 depletion. This suggests that Plk1 mainly affects origin activation and fork speed in the genomic regions governed by the slow process. Independently of DNA combing experiments and consistent with the important role of Plk1 during the S phase, we found a significant increase (+18%) in the time to reach near complete replication using quantification of ${}^{32}{\mathbf {P}}$-dATP incorporation (Supplementary Figure S11A and B). Since no delay in S phase entry was observed in the absence of Plk1 (46), this suggests that it is the duration of S phase that is increased.

We conclude that Plk1 depletion has the strongest effect on separating the two replication processes highlighted by RepliCorr analysis and that Plk1 regulates the spatial organization of origin firing and fork progression along the genome.

## Discussion

In this study, we have explored how the activation of replication origins is coordinated along the chromosomes in a vertebrate model system. To address this question, we first developed a novel analysis method describing the spatial replication pattern of stretched single DNA molecules obtained by DNA combing or optical mapping after replication in the *Xenopus in vitro* system. We classified the similarity of these patterns, taking advantage of the correlation concept, and called this analysis method ‘RepliCorr’. Second, our results reveal that two independent, spatiotemporally exclusive processes regulate DNA replication in *Xenopus*. These processes differ by their replication fork speed and rate of origin firing. Third, the abrogation of two main regulatory pathways of the DNA replication program, the replication checkpoint and Rif1 had either no or only a moderate influence on the spatial distribution of these processes. However, the depletion of the Plk1, known as a checkpoint adaptor, abolished the spatial separation of these processes. Thus, our results suggest that Plk1 is an important coordinator of the spatial replication program and the initiation–elongation coupling along the chromosomes in *Xenopus*.

### The replication dynamics can be described as a combination of only two independent processes with distinct fork speeds and initiation rates in *Xenopus*

To analyze the dynamics of the replication process, detecting replicated tracks on individual DNA molecules allowed us to measure the time-dependent rate of DNA replication. In past studies, only initiation rates have been used to explore the replication process quantitatively. We previously reported that the initiation rate follows a typical bell-shaped curve during the S phase in several model organisms (8–10). Since apparent fork speeds are not necessarily constant throughout the S phase (1,15,5), we have now investigated how initiation and elongation are quantitatively connected to ensure S phase completion. Using RepliCorr, we show that the replication profiles of single DNA molecules during the normal S phase in *Xenopus* can be described by either of two processes, specified by the inverse relationship between initiation rate and fork speed. This confirms that initiation rate and fork speed are intimately linked properties of undisturbed DNA replication, as observed in mammalian cells (15). Unexpectedly, we further demonstrate that the observed replication patterns can be described by a linear combination of two extreme configurations: one with a low initiation rate coupled with a fast fork progression and one with a high initiation rate associated with a slow fork progression (Figure 6B, left panel). We observe these two replication modes at the level of DNA molecules with 80–150 kb of size, corresponding to the size of replication clusters previously described in the *Xenopus in vitro* system (3,5). Therefore, the two replication modes may characterize two replication cluster types whose possible differences in chromatin structure or looping would be interesting to investigate.

Are these two different replication strategies in the *Xenopus in vitro* system correlated with the temporal program? From the RepliCorr analysis of the HOMARD experiments, we conclude that both processes co-exist in genomic sequences replicating during the early S phase, whereas the slow process is predominant in some genomic sequences replicating in the late S phase. The change from a low to a higher initiation rate could be explained by initially limiting initiation factors, which, as the S phase progresses, are recycled toward origins to be activated in the unreplicated fraction of the genome. Fork speed could slow down during the S phase because of the progressive exhaustion of replication factors such as dNTPs at the nuclei concentration we used in the *in vitro* system and a low ribonucleotide reductase (RNR) activity expression. In early *Drosophila* embryos, the maternally deposited RNR is activated as dATP concentration decreases during the S phase (56). However, fibers with slow process patterns were also observed in the early S phase in DNA combing and HOMARD experiments, and decreasing fork speed in *Xenopus* was observed at a 10 times lower nuclei concentration (5), arguing against the exhaustion of replication factors. Slow or stalled replication forks are considered a sign of replication stress, which may result from DNA damage. Still, our observations suggest that impairment of the DNA replication checkpoint does not affect dual DNA replication modes in *Xenopus*. Finally, chromatin assembly can also regulate fork speed (57). It is possible that the chromatin remodeling of sperm nuclei introduced into egg extracts creates a heterogeneous chromatin with co-existing accessible and difficult-to-replicate regions without activation of checkpoint mechanisms during DNA replication. Interestingly, we have shown that the ribosomal DNA repeat domain replicates later and has a more nuclease-resistant structure than the bulk genomic sequences in egg extracts (58). More recently, it was reported that a homogeneously methylated fraction of histone H3 is retained in spermiogenesis, during early *Xenopus laevis* development, and in sperm nuclei replicating in egg extracts, marking regulatory sequences for developmental genes (59). It is tempting to speculate that these different paternal epigenetic marks with repressive H3 (H3K27me3) and active H3 (H3K4me3) methylation could partly be responsible for the observed different replication modes.

### Plk1 regulates the spatial replication program

RepliCorr analysis of single-molecule replication patterns after targeting three different pathways of the replication program revealed that only Plk1 depletion strongly affected the pattern distributions between the fast and slow process. Without Plk1, these two processes were no longer independent. Still, they coexisted on DNA molecules with a high degree of replication, suggesting that Plk1 depletion canceled the spatiotemporal exclusive character of the two processes and changed the dynamics of the slow process rather than the fast process (Figure 6B, right panel). In contrast, Rif1 depletion or Chk1 inhibition and its overexpression only moderately modified the pattern distribution and the two processes. This is consistent with the fact that Plk1 depletion induced significant changes in initiation rates, IODs and eye lengths when molecules of the same replicated fraction were compared (47), whereas Rif1 depletion (38) or Chk1 inhibition (31) did not. Therefore, these findings suggest that Plk1 promotes origin activation inside replication clusters. In contrast, Rif1 has been shown to mainly accelerate both whole cluster activation and replication of larger replication domains in *Xenopus* (38) and mammalian cells (36,37) consistent with only a small effect of Rif1 depletion on RepliCorr patterns detected in this study. Interestingly, our results suggest that Plk1 mainly supports the slow process that predominates during the late S phase. Therefore, Plk1 may favor the late origin firing, similar to the dispersed, late firing origins identified in other eukaryotes (60–63). Chk1 inhibition or Rif1 depletion increases the initiation frequency of the slow process, albeit to a much lesser extent than Plk1, suggesting that the spatial regulation of the replication program by these known regulators mainly occurs via the regulation of the slow process in addition to their effects on the temporal program (47,38).

It is unclear what could be the molecular mechanisms of how Plk1 locally regulates both fork speed and initiation rate in some genomic regions but not in others. Recently, we demonstrated that Plk1 could inhibit the Chk1-dependent replication checkpoint (46) and could phosphorylate the PP1 binding site of Rif1, which prevented PP1 inhibition by Rif1 in *Xenopus* (47). However, we observed only modest effects on replication pattern distribution after Chk1 inhibition, overexpression or after Rif1 depletion. Therefore, other Plk1-dependent pathways seem to be necessary for this local regulation. Interestingly, Plk1 co-immunoprecipitated with pre-initiation complex proteins Treslin, MTBP and TopBP1 (47), which are rate limiting for replication in *Xenopus* and budding yeast (64,65). In addition, Plk1 depletion results in a longer persistence of these factors on chromatin, and it has been suggested that these factors should dissociate from activated origins to allow fork elongation (conversion from pre-initiation complex to Cdc45-MCM-GINS complex) (66,67). It is, therefore, tempting to speculate that in the absence of Plk1, the recycling of Treslin/MTBP and TopBP1 toward neighboring origins is slowed down, resulting in a decrease in the initiation rate. In further support of this hypothesis, a recent study showed that for dormant origin firing, the linear correlation between IODs and fork speed at different concentrations of aphidicolin is also dependent on TopBP1 but not on Chk1 (68). Another possibility is that Plk1 directly interacts with replication fork proteins to reduce fork speed. In support of that hypothesis, we have shown that Plk1 co-immunoprecipitates in chromatin fractions with replisome components MCM2–7, RFC1–5 and AND-1, and the chromatin remodeler FACT subunit SSRP1 (47). The latter two are known to be implicated in the regulation of fork rate (69,70). Since the phosphorylations of MCM6–7, RFC1, AND-1/WHDH1, and SSRP1 are sensitive to a Plk1 inhibitor in mammalian cells (71), it would be interesting to dissect whether Plk1 could affect the activity of these fork proteins in *Xenopus*. During the very early stages of *Xenopus* development, Plk1 levels are high but decline after the onset of zygotic transcription after the mid-blastula transition (MBT) (46). The number of active origins also declined after the MBT (72,73), but fork speed was not determined. During early mice developmental stages, mean origin distances gradually increase after the two-cell embryo stage together with mean fork speed (24). It would be interesting to investigate whether Plk1 could also be implicated in changing the dual replication modes during development.

In conclusion, our work shows that Plk1 promotes the spatiotemporal heterogeneity of initiation rate and fork speed in *Xenopus*. Plk1 is often overexpressed in aggressive cancer types (74) but is mainly studied for its role in mitosis entry. We believe it is necessary to consider the role of Plk1 during the S phase more carefully in tumor development. RepliCorr is a powerful tool for analyzing replication dynamics and initiating rate and fork speed coupling in the naturally synchronous *Xenopus in vitro* system. It is robust enough to handle fluctuations resulting from the stochasticity of the replication process. The bell shape of the initiation rate was first observed in *Xenopus* (8) and later found to be universal in all eukaryotes (9,10). We extended the application of RepliCorr to DNA combing data from *S. cerevisiae* (75), calculating initiation frequencies and auto-correlation profiles. Our analysis revealed that these profiles could be fitted with a single- or dual-process model (Supplementary Figure S12). Looking for a minimal description of the replication process, the single-process model appears sufficient to describe the spatial replication patterns in budding yeast. Further studies are required to elucidate the molecular basis underlying these differences between the two model systems.

The extension to another model system revealed certain limitations of RepliCorr. The method relies on continuous replication labeling from synchronous nuclei or cells sampled at different times during the S phase to comprehensively analyze the replication dynamics. Additionally, RepliCorr is restricted to analyzing the increasing portion of the initiation frequency curve corresponding to fibers with a maximal replication extent of 75% in the *Xenopusin vitro* system and around 50% in budding yeast. Another limitation of this study is the inability to determine whether the two independent replication modes identified in *Xenopus* correspond to specific genomic sequences. This limitation stems from the incomplete assembly of the *X. laevis* reference genome, the sequence differences between our *Xenopus* strain and the reference strain, or other unknown technical issues, which precluded the integration of sequence data in our optical mapping experiments. Future research should explore the applicability of RepliCorr to single-molecule replication data from other multicellular models as they become available. Such studies could further validate its robustness and provide deeper insights into the universality of replication dynamics across diverse biological systems.

## Supplementary Material

gkaf007_Supplemental_File

## Data Availability

The DNA combing datasets and HOMARD data analyzed during this study are available from the corresponding author on request. The code for the RepliCorr analysis is deposited on GitHub (https://github.com/DidiCi/RepliCorr) and figshare (https://doi.org/10.6084/m9.figshare.28091093.v1).

## Notes

Present address: Francesco de Carli, Corporate Preclinical R&D, Chiesi Farmaceutici SpA, Parme, Italy.
