# Supplementary material for: Dual DNA replication modes: varying fork speeds and initiation rates within the spatial replication program in Xenopus
Source: Nucleic Acids Res. 2025 Jan 30;53(3):gkaf007. doi: 10.1093/nar/gkaf007 (PMC11781033; doi:10.1093/nar/gkaf007)
Supplement: gkaf007_Supplemental_File [file gkaf007_supplemental_file.pdf]

Dual DNA Replication Modes: Varying fork speeds and initiation rates within the spatial replication program in *Xenopus*

D. Ciardo, O. Haccard, F. de Carli, O. Hyrien, A. Goldar, K. Marheineke

Supplementary Data

-Supplementary Methods

-Supplementary Figures S1-12 and Tables S1-3

Supplementary Methods

The theoretical framework

Stable phase fraction

The quantitative relation between the density of nucleated domains, the speed of growth, and the transformed volume during a nucleation and growth process were obtained between the end of the 30s and the beginning of the 40s by Kolmogorov, Johnson, Mehl and Avrami. The theory was pushed further by Sekimoto for the one-dimensional case [11]. In the KJMA model, the system undergoes a gradual transformation from an initial phase (metastable phase) to a final phase (stable phase), and the two phases coexist during the entire transition. In our case, the two phases correspond to the unreplicated and replicated state. During the transformation, stable domains nucleate randomly and grow in the metastable phase. The critical nucleus size, above which nuclei grow but below which they dissolve, is considered in the replication process to be infinitesimal. The process is characterized by $I(t)$, the rate of nucleation per unit volume of metastable material, and $2v$, the constant positive speed at which the stable phase grows after nucleation. We introduce the phase indicator function $u(r,t)$, defined as follows:

$$u(x,t) = \begin{cases} 1, & \text{if the point r belongs to the metastable phase} \\ 0, & \text{otherwise} \end{cases} \tag{1}$$

The fraction $\phi(t)$ for the metastable phase is then defined as:

$$\phi(t) = \langle u(x,t) \rangle \tag{2}$$

where $\langle\rangle$ denotes the average over the ensemble of the random variable $u(x,t)$. $\phi(t)$ should be a decreasing function of t.
In order to obtain the formula for the metastable phase fraction, we must introduce the notion of a causal cone. This notion allows us to keep track of the complete history of the nucleation process, which is necessary in the case of continuous nucleation. The growth of the domain with constant speed $2v$, from a specific nucleation site, can be viewed as an expanding triangle in the space-time representation. For multiple nucleations, the growth can be represented as the combination of different triangles, as shown in Fig. 1.

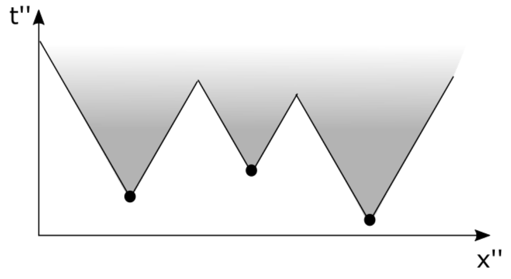

Fig. 1. Nucleation and growth in one dimension. The stable domains that grow from multiple nucleation sites are unions of triangles in the space-time representation; the resulting region is highlighted in grey.

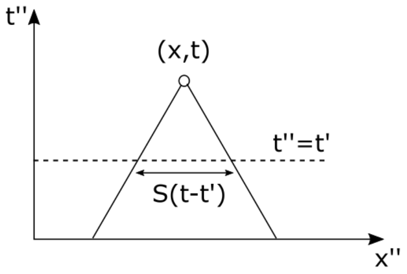

Fig. 2. Representation of the one dimensional causal cone for the point (x,t).

On the other hand, for a point x to remain in the metastable phase at time t, nucleation events cannot occur within the inverted triangle, whose apex is at the point (x,t), as shown in Fig. 2. The inverted triangle is called the causal cone. As $\phi(t)$ corresponds to the probability that for any $t' < t$ nucleation centers do not appear in the length $S(t-t') = 2v(t-t')$. Assuming nucleation as a rare event with a density $I(t)$, we use the Poisson distribution to write:

$$\phi(t) = exp\Big(-\int_0^{\infty} dt' I(t') S(t-t')\Big) \tag{3}$$

with $S(t-t') = 0$ for $t < t'$. This expression and what follows are valid only if: i) the speed v is not an increasing function of t, ii) the rate of nucleation is spatially homogeneous, and iii) the nucleation events occur independently. The fraction $f(t)$ for the stable phase is:

$$f(t) = 1 - \phi(t) \tag{4}$$

To have a more complete description of the nucleation and growth process, it is possible to analyze other quantities such as the length of islands, holes, and the island-to-island distances. The probability distribution of these quantities can be expressed as a function of the time t or the fraction of the stable phase f. In his work, Sekimoto also studied the time evolution of domain statistics by solving Fokker-Plank-type equations for island and hole distributions in the case of a constant nucleation rate I(t)=const [9, 10]. Sekimoto's approach was extended in [6] in the case of a general nucleation rate I(t).

The correlation function

A further development of the theory was achieved by Sekimoto [11], who derived an exact expression for the two-point correlation function of growing domains in different dimensions. The correlation function provides in fact a more complete characterization of the spatial distribution of the two phases. Otha et al. [8] extended this result, introducing the possibility of nucleation of p different stable phases, and the limit of $p \to \infty$ was analyzed by Axe and Yamada in one and two dimensions [4].
We derive the two-point correlation function in one dimension as deduced by Sekimoto [11] and Ohta, Ohta and Kawasaki [8]. The two-point correlation function can be expressed as:

$$G(r,t) = \langle u(x,t)u(x+r,t) \rangle \tag{5}$$

which quantifies the probability that two points separated by a distance r are both in the metastable phase at time t. In

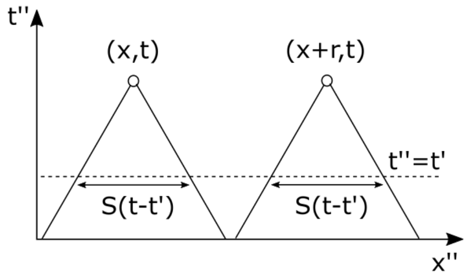

Fig. 3. Representation of the causal cones for two uncorrelated points.

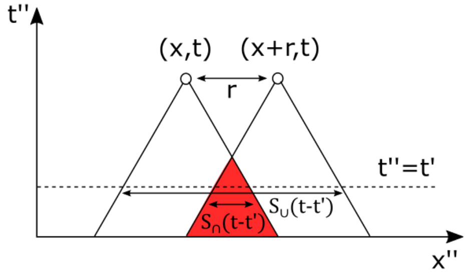

Fig. 4. Representation of the causal cones for two correlated points. The region of overlap (in red) defines the degree of correlation between the two points.

this case, the probability is governed by the union of the causal cones relatives to the two points (Fig. 3 and Fig. 4).

With the same argument that we used for the evaluation of $\phi(t)$, we can write:

$$G(r,t) = exp\Big(- \int_0^\infty dt' I(t') S_\cup(t-t') \Big) \tag{6}$$

where $S_\cup(t-t')$ is the spatial length, in which no nucleation events must occur at time $t' < t$ in order that the points x and $x+r$ belong to the metastable phase at time t. We have:

$$S_\cup(t-t') = 2S(t-t') - S_\cap(t-t') \tag{7}$$

where $S_\cap(t-t') = 2v(t-t') - r$ is the spatial length relative to the eventual intersection of the two causal cones and is equal to zero for $r > 2v(t-t')$. By substituting (7) in (6), we obtain:

$$\begin{aligned} G(r,t) =& exp\Big(- \int_0^\infty dt' I(t') 2S(t-t') \Big) exp\Big(\int_0^\infty dt' I(t') S_\cap(t-t') \Big) \\ =& \phi(t)^2 exp\Big(\int_0^\infty dt' I(t') S_\cap(t-t') \Big) \end{aligned} \tag{8}$$

For $r > 2vt$, $G(r,t) = \phi(t)^2$ meaning that the phase state of two points at distance $r > 2vt$ are independent. This reflects the fact that at the time t, the maximum size of a stable domain is $2vt$ and no long-range correlation is mediated by the stable domains.

We can then easily obtain the two-point correlation function for the stable phase as:

$$\begin{aligned} C(r,t) &= \langle (1-u(x,t))(1-u(x+r,t)) \rangle \\ &= 1 - \langle u(x,t) \rangle - \langle u(x+r,t) \rangle + \langle u(x,t) u(x+r,t) \rangle \\ &= 1 - 2\phi(t) + G(r,t) \end{aligned} \tag{9}$$

Correlation function of fluorescence profiles from DNA fiber experiments

As detailed above, Kolmogorov, Johnsol, Mehl and Avrami developed a stochastic model that describes the kinetics of the transition from an initial phase (metastable phase) to a final phase (stable phase). [7, 5, 1, 2, 3]. A further development of the theory was achieved by Sekimoto [11], who derived an exact expression for the two-point correlation function of growing domains in different dimensions. The KJMA theory can be used to describe the replication process if the replicated state is considered as the stable phase and the unreplicated phase as the metastable phase. In this context, the growth speed v corresponds to the replication fork speed, and the nucleation rate $I(t)$ to the frequency of initiation. Once obtained an explicit form for the two-point correlation function, we applied it to the study of the correlation function of fluorescence intensity profiles from DNA fiber experiments.

In order to use the expressions (4) and (9), we need to choose an explicit form for the frequency of initiation $I(t)$. We will consider the form:

$$I(t) = I_0 t^\alpha \tag{10}$$

with $I_0 \geq 0$ and $\alpha \geq 0$. This expression is a good approximation for the increasing region of the frequency of initiation. We then restricted the analysis to this region. By using the Eq. (3) and (4), we obtain:

$$\begin{aligned} f(t) =& 1 - exp\Big(- 2vI_0 \int_0^t dt' (t')^\alpha (t-t') \Big) \\ =& 1 - exp\Big(- \frac{2vI_0 t^{\alpha+2}}{(\alpha+1)(\alpha+2)} \Big) \end{aligned} \tag{11}$$

where we used $S(t-t') = 0$ for $t < t'$. In a similar way, from the Eq. (8) and (9), we have:

$$\begin{aligned} C(r,t) &= 1 - 2\phi(t) + \phi(t)^2 exp\Big(I_0 \int_0^{t-r/2v} dt' (t')^\alpha [2v(t-t') - r] \Big) \\ &= 1 - 2\phi(t) + \phi(t)^2 exp\Big(\frac{2vI_0 t^{\alpha+2}}{(\alpha+1)(\alpha+2)} \Big(1 - \frac{r}{2vt} \Big)^{\alpha+2} \Big) \end{aligned} \tag{12}$$

where we used $S_\cap(t-t') = 0$ for $r > 2v(t-t')$ or equivalently for $t' > t - \frac{r}{2v}$. The Eq. (12) will be valid for $r < l_{max} = 2vt$, where l_{max} represents the maximum replication eye length present at time t.

Statement of the problem

The DNA combing and HOMARD technique allows the analysis of the replication state of a DNA fiber at a certain time during the replicative phase. Between all the information that we can obtain through the analysis of the fluorescence intensity profiles, we will focus on the replicated fraction, the frequency of initiation, and the correlation function of the single fiber. In the analysis, there will be two major consequences related to the use of data obtained with the DNA combing technique:

1. The finite size of the analyzed fibers implies that we only have access to the local evolution of the process. The results of the quantitative analysis will be not valid for the replication process of the entire genome.
2. To apply the theory as it is, we would need to know the exact time at which replication starts on the single fiber, but the experimental technique does not provide this information. We will have to use the replicated fraction as

a measure of the local evolution of the process. This will introduce some uncertainty in the analysis, due to the lack of knowledge of the hidden variable, that is, the time.

We can define the problem as follows. We want to analyze the similarity between replication patterns of different fibers by comparing the correlation function of the fluorescence intensity profiles. The specific pattern depends on the frequency of initiation and the fork speed. So, we will estimate the variables v, I_0 and α, given the experimental frequency of initiation $I(f)$ as a function of the replicated fraction f and the correlation function $C(r, f)$ for different replicated fractions f as a function of r. The time will be obtained from the analytical inversion of the Eq. (11) as $t = f^{-1}(v, I_0, \alpha)$.

References

1. Melvin Avrami. Kinetics of Phase Change. I General Theory. *The Journal of Chemical Physics*, 7(12):1103–1112, December 1939.
2. Melvin Avrami. Kinetics of Phase Change. II Transformation-Time Relations for Random Distribution of Nuclei. *The Journal of Chemical Physics*, 8(2):212–224, February 1940.
3. Melvin Avrami. Granulation, Phase Change, and Microstructure Kinetics of Phase Change. III. *The Journal of Chemical Physics*, 9(2):177–184, February 1941.
4. J. D. Axe and Y. Yamada. Scaling relations for grain autocorrelation functions during nucleation and growth. *Physical Review B*, 34(3):1599–1606, August 1986.
5. W. A. Johnson and R. F. Mehl. Reaction Kinetics in Processes of Nucleation and Growth. In *Trans. A.I.M.E.*, volume 135, page 416, 1939.
6. Suckjoon Jun, Haiyang Zhang, and John Bechhoefer. Nucleation and growth in one dimension. I. The generalized Kolmogorov-Johnson-Mehl-Avrami model. *Physical Review E*, 71(1), January 2005. Number: 1.
7. A.N. Kolmogorov. A statistical theory for the recrystallisation of metals, Akad Nauk SSSR, Izv. *Izv. Akad. Nauk. SSSR*, 3, 1937.
8. Shigetoshi Ohta, Takao Ohta, and Kyozi Kawasaki. Domain growth in systems with multiple-degenerate ground states. *Physica A: Statistical Mechanics and its Applications*, 140(3):478 – 505, 1987.
9. Ken Sekimoto. Kinetics of magnetization switching in a 1-D system II-long time behavior of switched domains. *Physica A: Statistical Mechanics and its Applications*, 128(1):132 – 149, 1984.
10. Ken Sekimoto. Kinetics of magnetization switching in a 1-D system-size distribution of unswitched domains. *Physica A: Statistical Mechanics and its Applications*, 125(1):261 – 269, 1984.
11. Ken Sekimoto. Evolution of the domain structure during the nucleation-and-growth process with non-conserved order parameter. *Physica A: Statistical Mechanics and its Applications*, 135(2-3):328–346, 1986.

Supplementary Figures and Tables

	v (kb/min)	I_0 ($1/(kb*min^{\alpha+1})$)	α	χ^2
Simulation parameters	1	0.03	0	
Fit parameters<br>Simulated replication data Fig. 1C	1.009	0.029	0	2.2
Fit parameters<br>Experimental data Fig. 1D	0.76	0.000029	1.98	13.1

Supplementary Table S1: Comparison of fit results for correlation function with one process between simulated and experimental data. The rate of initiation is $I(t)=I_0t^{\alpha}$ per unit time per length of unreplicated DNA. Therefore, the replication process is characterized by three parameters: v, and for the initiation rate, I_0, α. Results of fit with a constant I_0, α and fork speed v for the autocorrelation function of simulated data from Figure 1C and from fit to experimental data from Figure 1D. The parameter values were averaged over 100 trials. A χ^2 value close to 1 is considered as a very good fit.

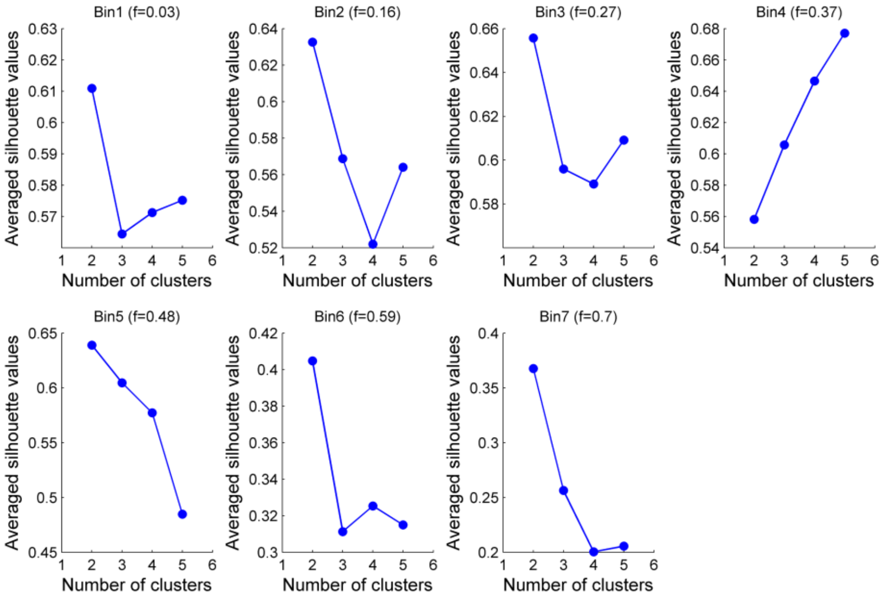

Supplementary Figure S1: Clustering evaluation by the average of silhouette values for the first control set. Each image corresponds to a different interval of replicated fractions from 0 to 75% (Bin 1-7) and the averaged replicated fraction is reported on the top. The fibers in each interval of replicated fraction were grouped into two to five clusters and the average silhouette value was calculated for each configuration. The silhouette value for a single fiber in a cluster measures how similar its correlation function is to the correlation functions of other fibers within the same cluster, compared to fibers in different clusters. The silhouette value ranges from -1 to +1. A value close to 1 indicates that the fiber's correlation function is much more similar to those within its own cluster than to those in other clusters. The average silhouette value across all fibers is then computed to assess the overall quality of the clustering. Mean C(r, f) profiles for molecules hierarchically classified into two clusters are reported in Fig. 2C.

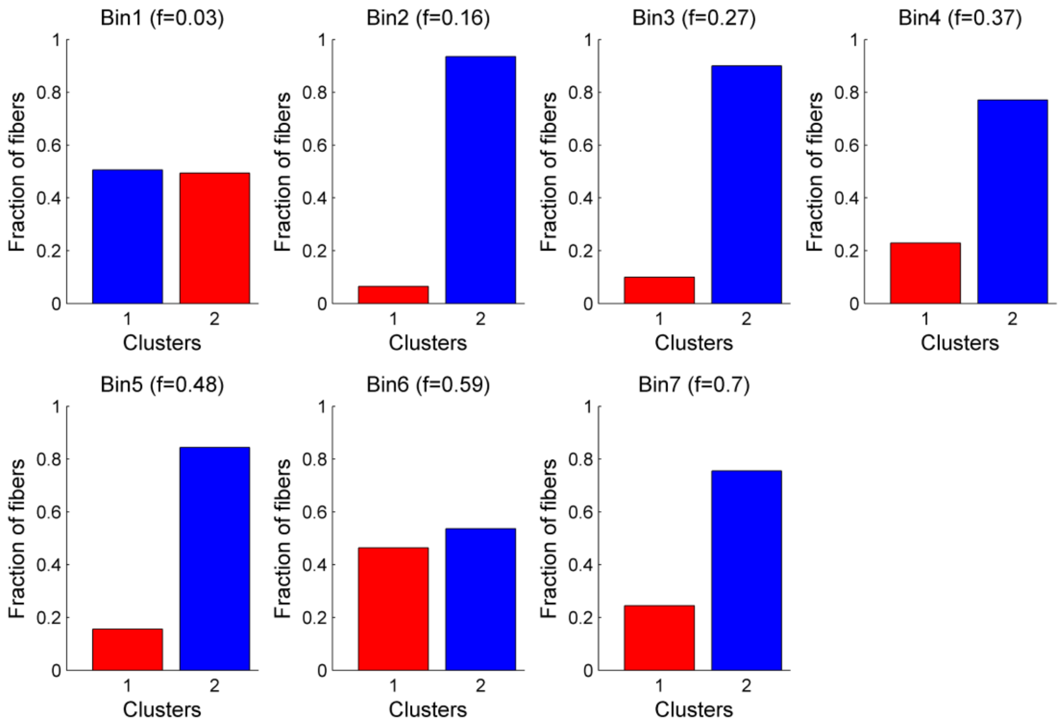

Supplementary Figure S2: Histogram reporting the fraction of fibers in each cluster for the first control set. Each image corresponds to a different interval of replicated fractions from 0 to 75% (Bin 1-7) and the averaged replicated fraction is reported on the top. Mean C(r, f) profiles for molecules hierarchically classified in the two represented clusters are reported in Fig. 2C.

Fit parameters	v_1 kb/min	I_1 1/(kb*min$^{\alpha+1}$)	α_1	T_{tot} 1	v_2 kb/min	I_2 1/(kb*min$^{\alpha+1}$)	α_2	$T_{tot\ 2}$	X^2
different *v* and I_0, α	1.07	0.002	0.003	23	0.17	0.016	0.24	19	0.75
different *v*, same I_0, α	1.4	**0.0013**	0.4	18	0.4	**0.0013**	0.4	**79**	**1.9**
same *v*, different I_0, α	**0.25**	0.0004	0.06	**103**	**0.25**	0.03	0.002	13	0.9

Supplementary Table S2: Fitting parameters values from Figures 3A with 2 processes with varying *v* and I_0 compared to fitting parameters with constant I_0, α, or constant *v* from Supplementary Figure S4 A and B. The initiation rate is given by $I(t)=I_0*t^{\alpha}$ per unit time per length of unreplicated DNA. Therefore, each process is characterized by three parameters: *v*, I_0, α. For different I(t) values of I_0 and α are set or parameters given after fitting. I_1 and I_2 are I_0 for process 1 and 2, respectively. T_{tot} is the time in min to replicate a fiber to 75 %. Values in red are those differing from the fit with two different I_0, α and *v*.

A

Different *v* and same I_0, α for the two processes

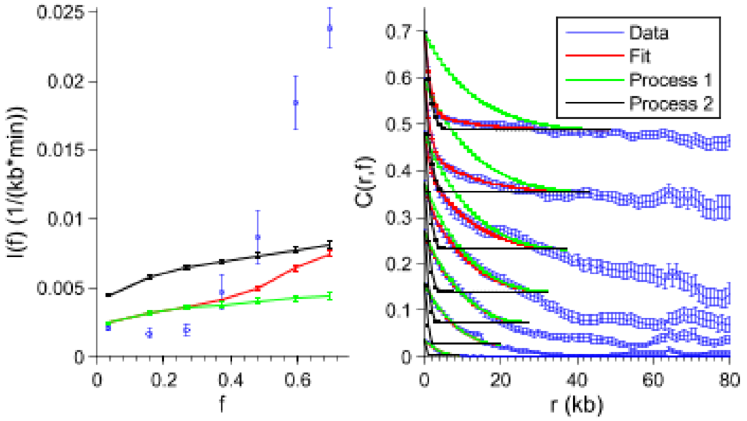

B

Same *v* and different I_0, α for the two processes

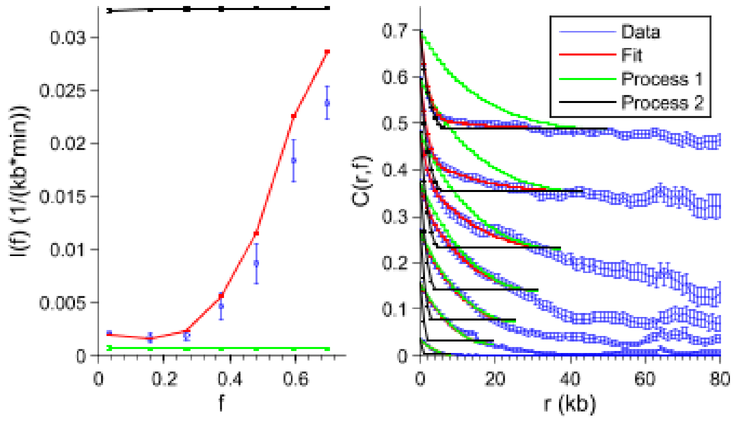

Supplementary Figure S3: Comparison between fits with two processes for correlation profiles with different or same I_0,α and *v*. (**A**) with different *v* and same I_0. (**B**) with same *v* and different I_0 for the two processes. (**C**) table with fitted parameter values from A and B compared to the parameters from Figure 3 A; with T_{tot} being the time in min to replicate a fiber to 75 %. We highlight values in red those differing from the fit with two different I_0 and *v*. The parameter values of fork speed and initiation frequency were averaged over 100 trials.

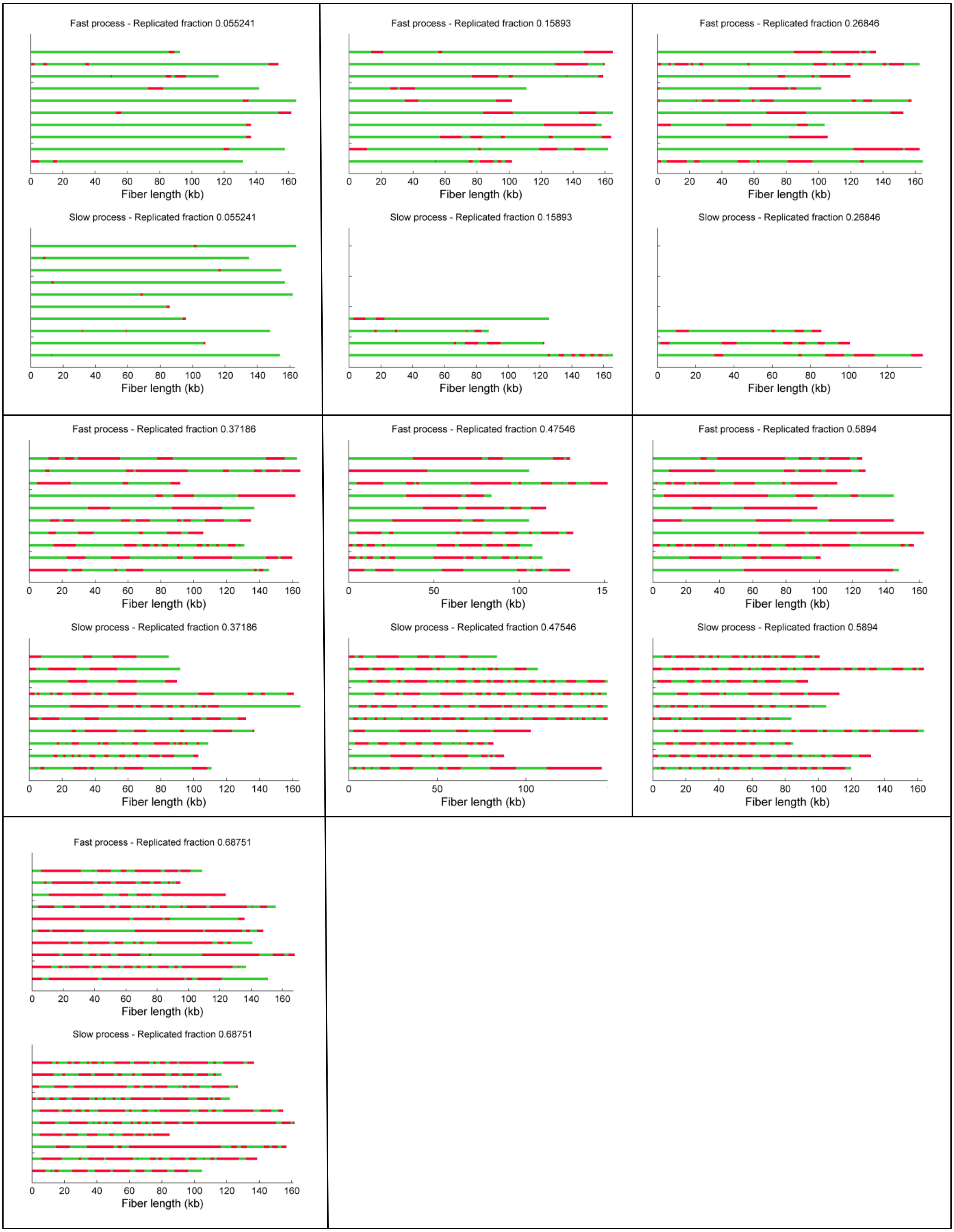

Supplementary Figure S4: Representative computer reconstructed fibers based on real fibers for the fast and slow process at the different replicated fractions for the control condition.

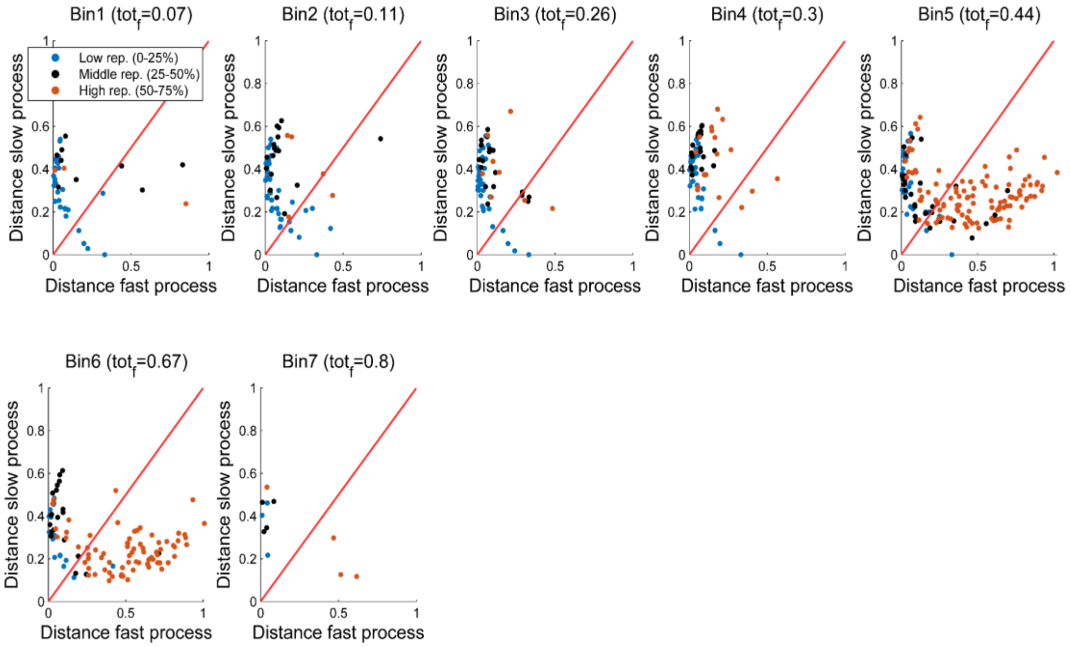

Supplementary Figure S5: Replicorr analysis per individual time point from three different experiments: Fibers from individual *in vitro* time points with similar mean replication extent compared to Figure 3B were analysed as in Figure 3B. Normalized correlation coefficients (ρ1, ρ2) between the molecule's *C(r,f)* and *C1(r,f)* and *C2(r,f)* were calculated. The similarity distance between the molecule and each process was defined as 1-ρ1 for the fast process 1 and 1- ρ2 for the slow process and represented on a two orthogonal axis plot. The red diagonal represents points of equal similarity to the two processes. Points above the diagonal are more similar to the fast process and points below are more similar to the slow process. Fibers with three different bins of replication inside each time point are shown in blue (0.5-25%), black (25-50%) and orange (50-75%). (45 min Replicate 1 (0.07), 50 min Replicate 2 (0.11), 55 min Replicate 1 (0.26) , (0 min Replicate 2 (0.3), 70 min replicate 3 (0.44), 85 min Replicate 3 (0,67), 100 min Replicate 3 (0.8)).

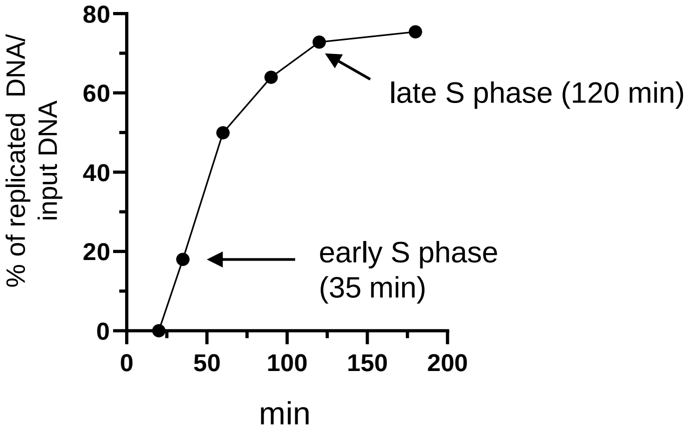

Supplementary Figure S6: Control ^{32}P-dATP incorporation kinetics for the HOMARD experiment: Sperm nuclei (1320 nuclei/µl), were incubated in the same egg extract than in the HOMARD experiment in the presence of ^{32}P-dATP and AF647-dUTP, reactions were stopped, DNA was precipitated as described (DeCarli *et al.,* 2017) and quantified as % of DNA synthesized per input DNA. Time points for the two equivalent incubation times (35, 120 min) analysed in HOMARD experiments are indicated.

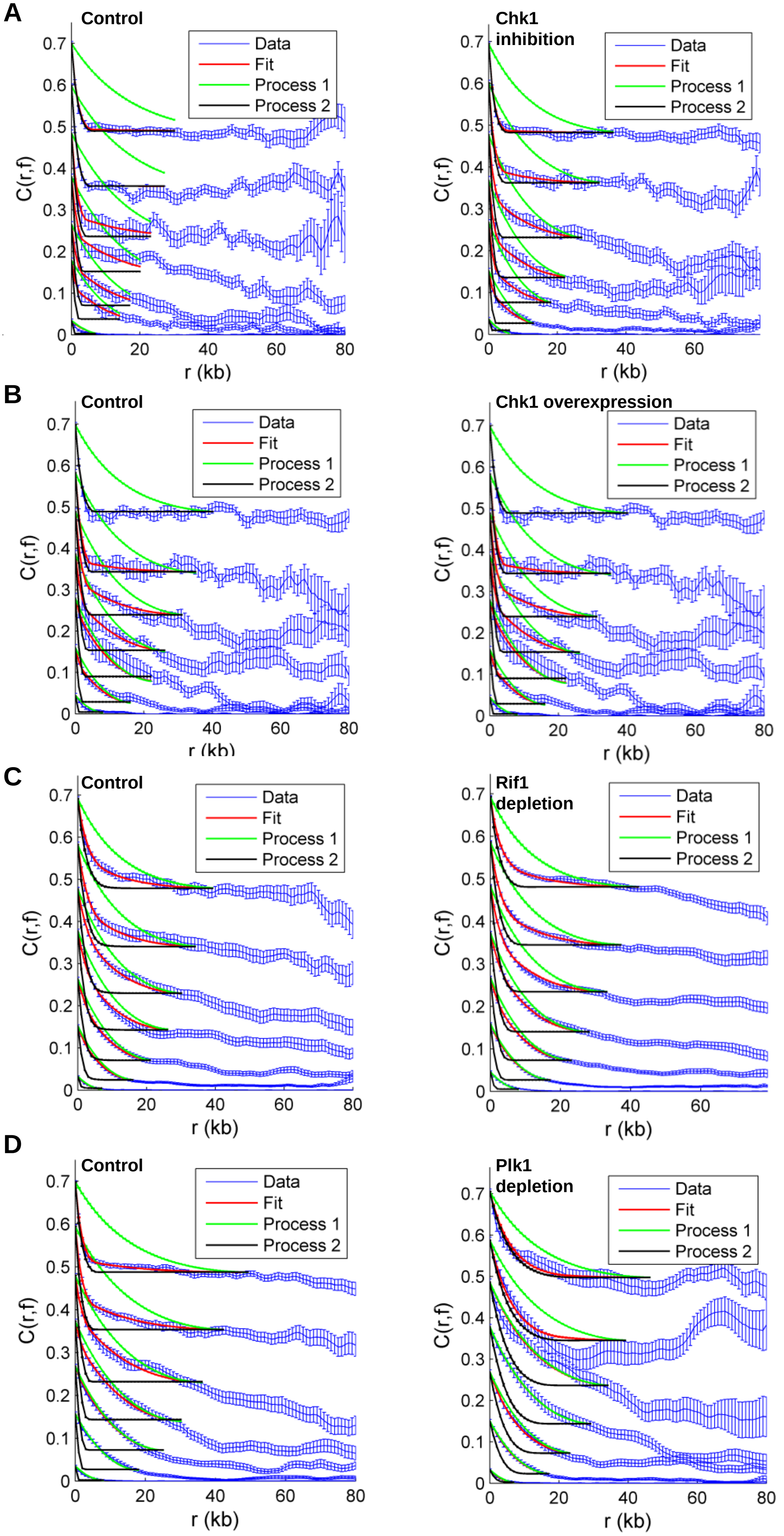

Supplementary Figure S7: Mean autocorrelation function *C(r,f)* profiles (blue curve, with standard deviation) with fit (red curve) as in Figure 3A for different pathways perturbations and corresponding controls. The green curve is the correlation profile produced by the fast fork process (*C1(r,f)*, model 1), and the black curve is the correlation profile produced by the slow fork process (*C2(r,f)*, model 2), f. (**A**) Chk1 inhibition by UCN-01 (n=2). (**B**) Chk1 overexpression (n=2). **(C)** Rif1 depletion (n=2). **(D)** Plk1 depletion (n=3).

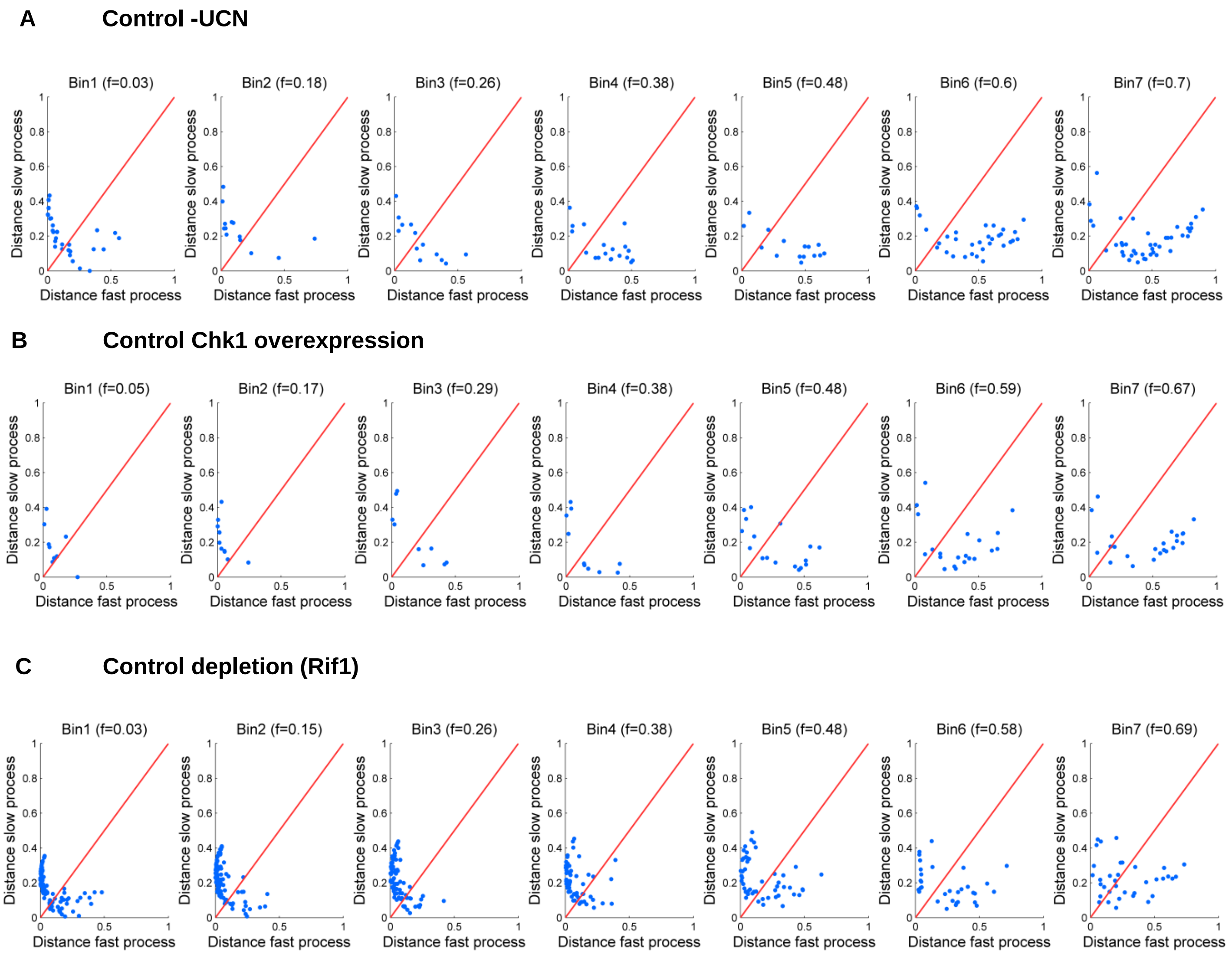

Supplementary Figure S8: Similarity distances of the fast and the slow process from control experiments from Figure 5. Normalized correlation coefficients (ϱ1, ϱ2) between the fiber's *C(r,f)* and $C_1(r,f)$ and $C_2(r,f)$ were calculated for control conditions in Figure 5 B-D. The similarity distance between the fiber and each process was defined as 1- ϱ1 for fast process and 1- ϱ2 for slow process and represented on a two orthogonal axis plot. The red diagonal represents points of equal similarity to the two processes. (**A**) +DMSO as control condition for Chk1 inhibition by UCN. (**B)** Control for Chk1 overexpression protein buffer addition. (**C**) Control depletion for Rif1 experiment.

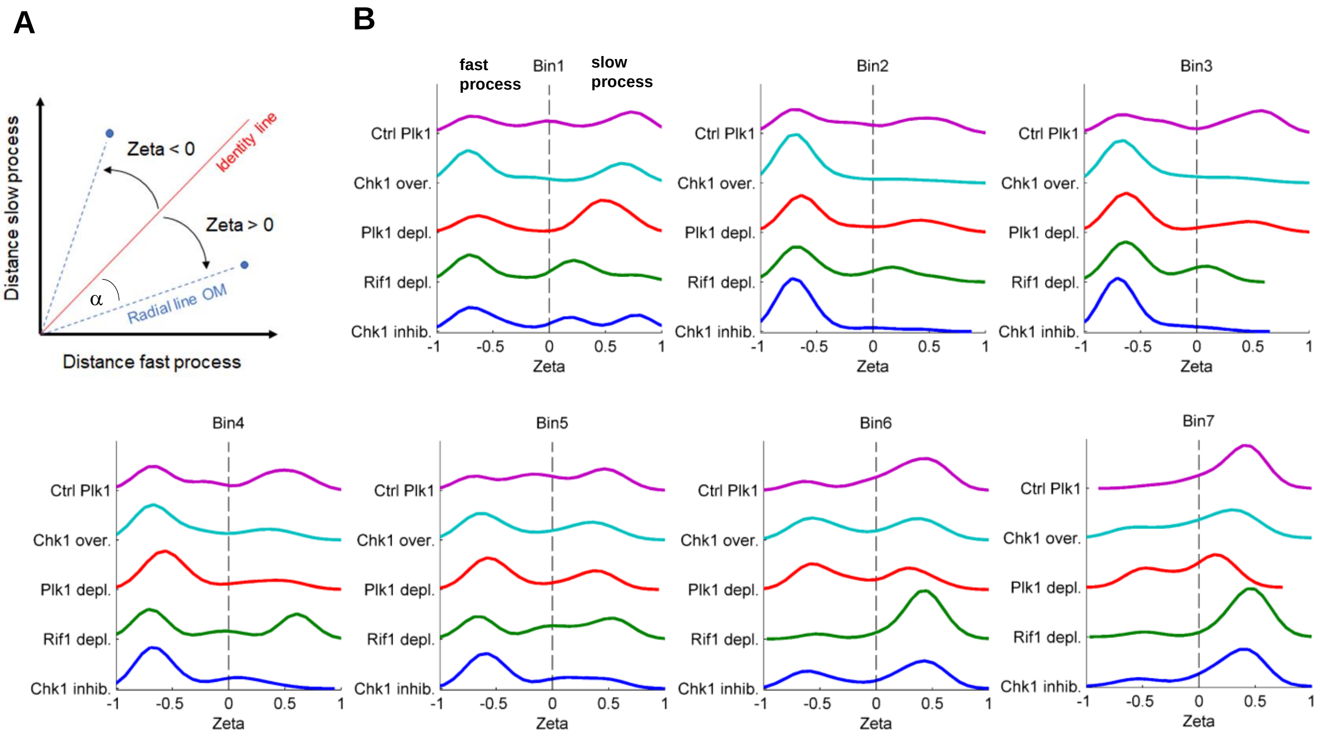

Supplementary Figure S9: **Comparative partitions of the replication process between slow and fast process for each replicated fraction under different conditions from Figure 5**. (**A)** Principle of the analysis: if the two processes have an equal influence on the replication of a fiber, then the fiber is represented by a point M that lies on the identity line of equation $y_M=x_M$. In other words, if the angle alpha between the x-axis and the radial line OM, connecting the origin (0,0) and the point M, is π/4, then the fast and slow process equiprobably influences the observed replication pattern. To have a measure of how each fiber deviates from this equiprobable influence scenario, we construct the variable zeta = π/4 - arctan(y_M/x_M), that is a measure how the radial line OM deviates from the identity line. To represent the distribution of observed zeta variables for a given sample and a specified bin of replicated fraction, we use the Kernel Density Estimation method to estimate the probability density function of the variable zeta based on the sample data. (**B)** We perform this analysis for all replication fraction bins and all conditions. Positive zeta values correspond to fibers where the slow process mainly drives the replication patterns, while negative zeta values reflect the preferential influence of the fast process. The Plk1 depletion condition is in the only condition where both processes are present at all replication fractions. Moreover, in the last replication bin, both peaks approach 0 after Plk1 depletion but not, or less, in the other conditions.

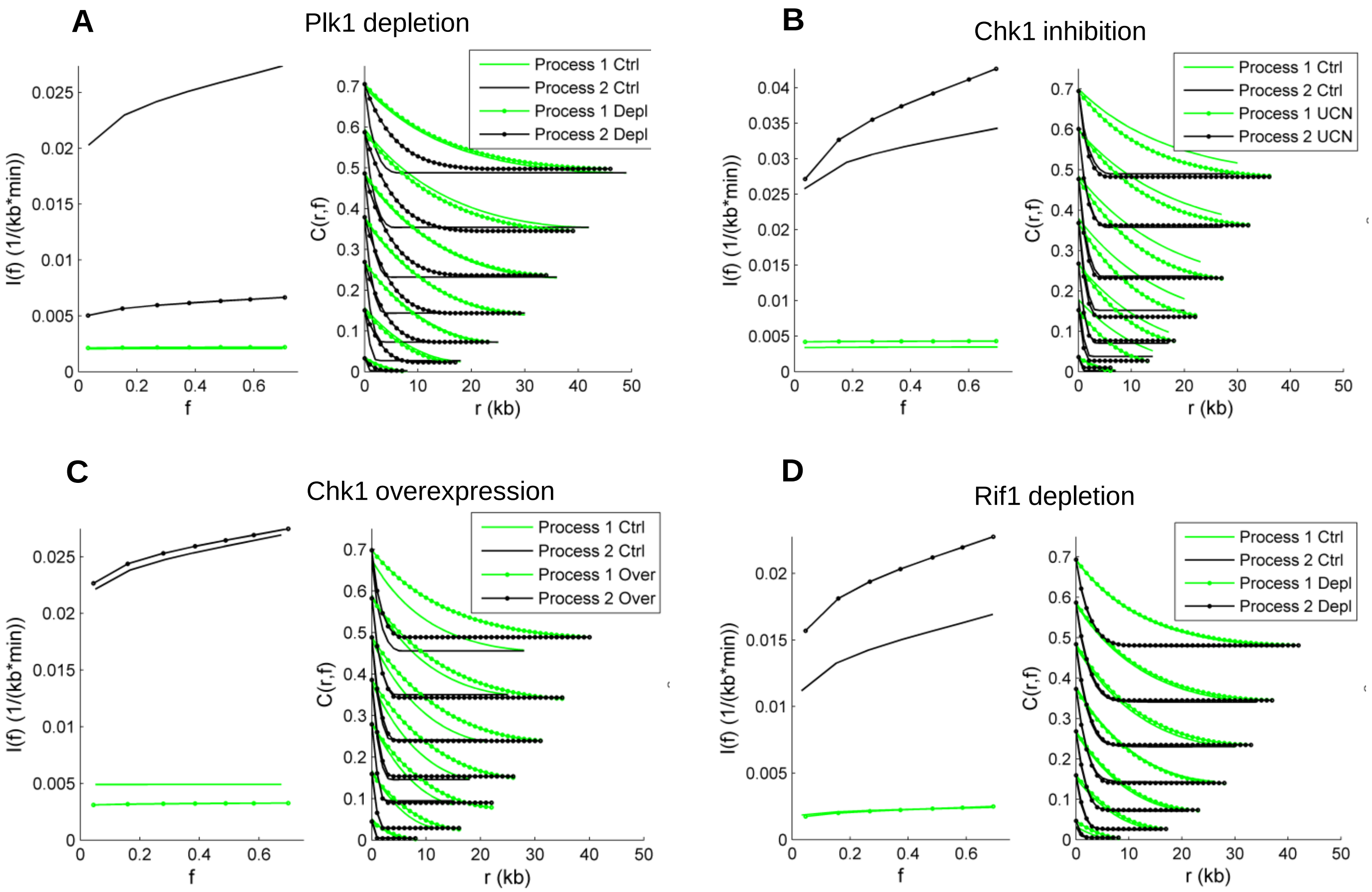

Supplementary Figure S10: Fits for correlation functions *C(r,f)* and associated initiation rates for fast process (process 1, green curves) and slow process (process 2, black curves) from experiments of (**A**) Plk1 depletion and its control. (**B**) UCN inhibition and control. (**C**) Chk1 overexpression and control. (**D**) Rif1 depletion and control.

	Fast process			Slow process			
Experimental conditions	***v_1*** **(kb/min)**	***I_1*** **$1/(kb*min^{\alpha+1})$**	***α_1***	***v_2*** **(kb/min)**	***I_2*** **$1/(kb*min^{\alpha+1})$**	***α_2***	**χ^2**
Control -UCN	2.251	0.003	0.009	0.211	0.021	0.197	1.14
Chk1 inhibition + UCN	1.556	0.004	0.014	0.191	0.020	0.356	0.40
Control Chk1 overexpression	1.141	0.005	0.002	0.138	0.018	0.178	0.63
+Chk1	1.333	0.003	0.032	0.167	0.019	0.155	0.68
Control depletion (Rif1)	0.807	0.002	0.160	0.208	0.008	0.383	0.86
Rif1 depletion	0.767	0.001	0.160	0.297	0.011	0.303	1.05
Control depletion (Plk1)	1.073	0.002	0.003	0.170	0.016	0.236	0.74
Plk1 depletion	1.034	0.002	0.022	0.471	0.004	0.209	0.93

Supplementary Table S3: Values from fits to autocorrelation profiles with two processes (1= fast process, 2=slow process) under different experimental conditions. The rate of initiation is $I(t)=I_0t^{\alpha}$ per unit time per length of unreplicated DNA. Therefore, the replication process is characterized by three parameters: *v,* and for the initiation rate, I_0, α. I_1 and I_2 are the I_0's for each process. The parameter values of fork speed and initiation frequency were averaged over 100 trials. A χ^2 value close to 1 is considered as a very good fit.

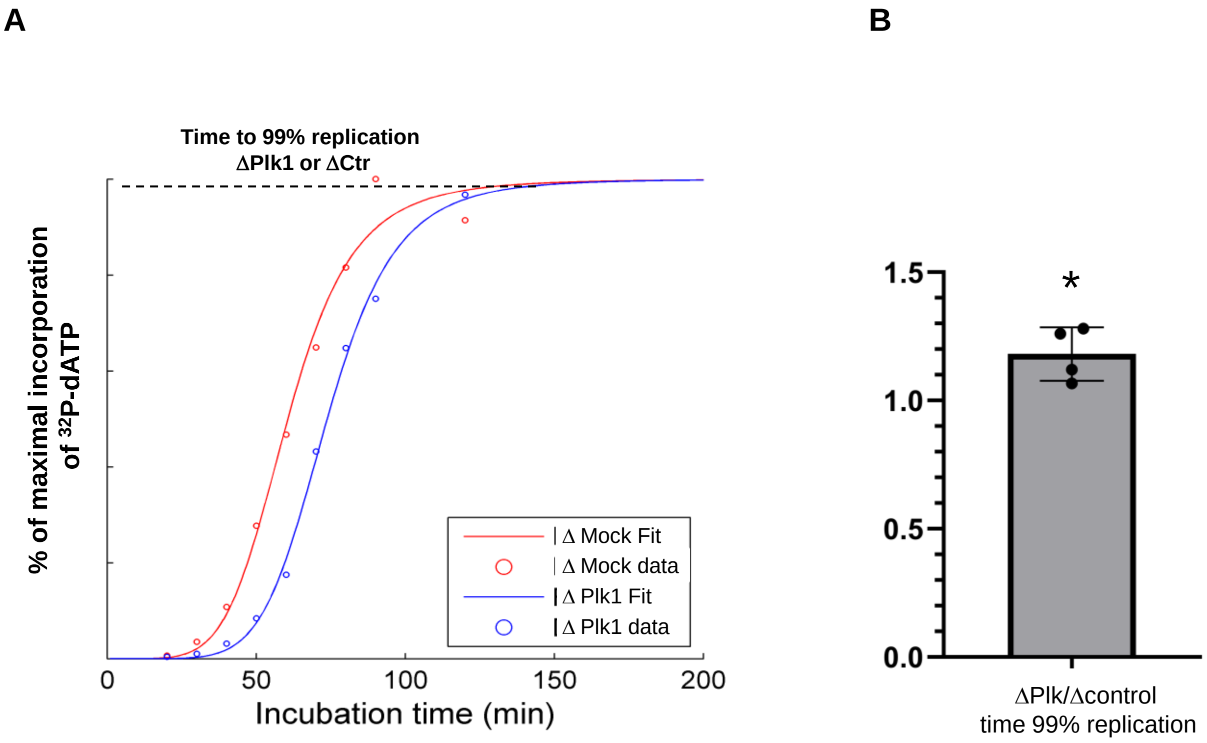

Supplementary Figure S11: Increase of time period to reach near 100 % replication after Plk1 depletion: (A) Sperm nuclei were incubated in control or Plk1-depleted egg extracts in the presence of α^{32}P-dATP, reactions were stopped at indicated times, DNA was purified and quantified as described in Ciardo *et al*., 2020. In order to compare different independent experiments, each experimental curve was independently fitted to a logistic function $\frac{1}{1+\exp(-b(x-c))}$ and normalised to the inferred maximum of incorporation for each experimental condition. The time period in min until 99% of incorporation was determined. A shift of 13 min in this period was found in the shown respresentative experiment. Similar calculated shifts were obtained at 95% or 50% of max. incorporation. (**B)** Mean ratio Plk1/control depletion time periods from 4 independent experiments (black points), one sample t-test, two tailed, $p=0.040$. In the absence of any detectable difference in S phase entry (Ciardo *et al.,* 2020), this observation suggests an increase in S phase length.

A

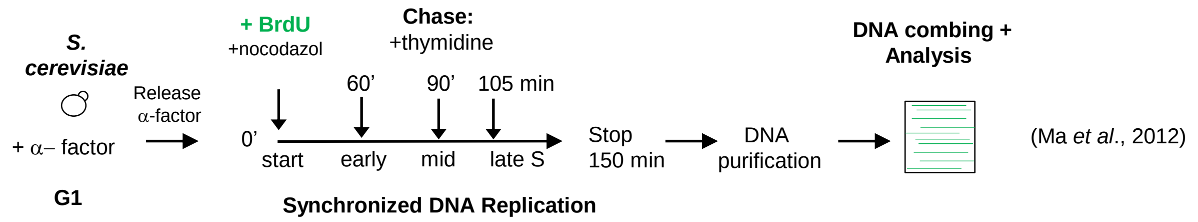

B C

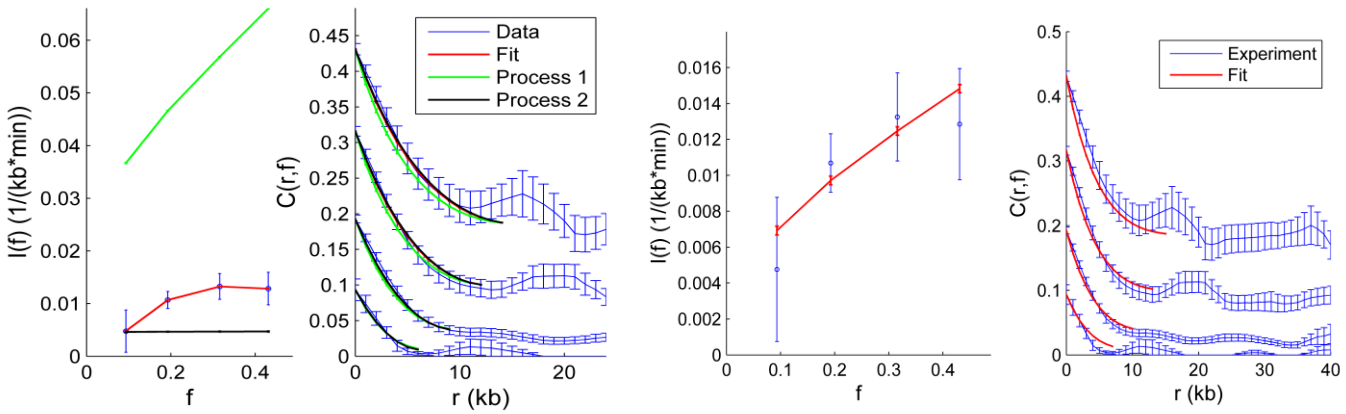

D

S. cerevisiae	v_1 (kb/min)	I_1 $1/(kb*min^{\alpha+1})$	α_1	v_2 (kb/min)	I_2 $1/(kb*min^{\alpha+1})$	α_2	NMSE	F	p-value
2 processes	2.291	0.0267	0.490	0.492	0.0045	0.0189	0.09	9.74	$7*10^{-7}$
1 process	0.614	0.000445	1.603	-	-	-	0.50		

E

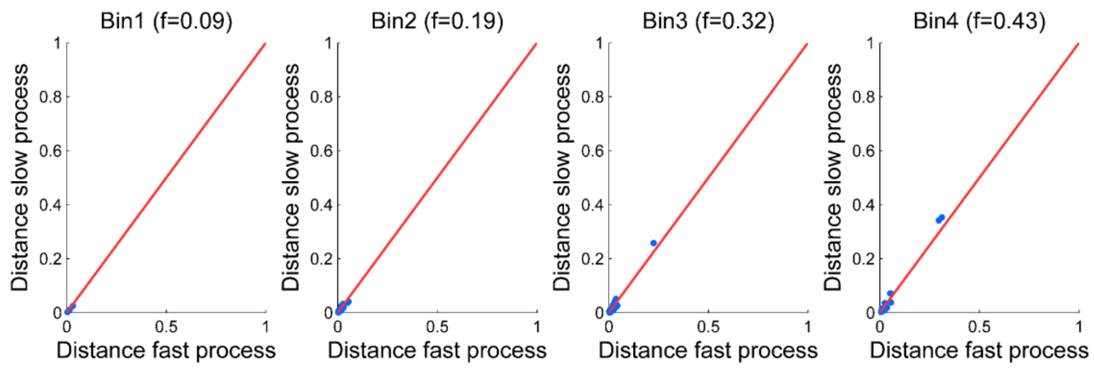

F

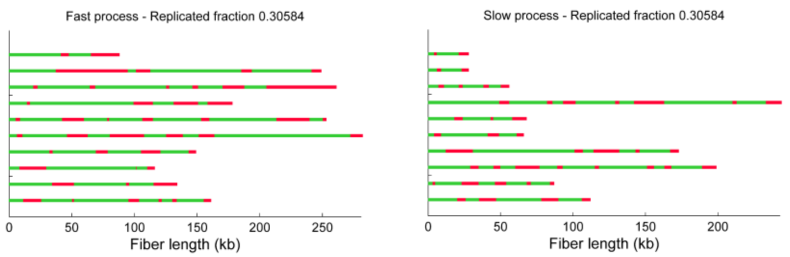

Supplementary Figure S12: Application of RepliCorr to DNA combing data from *S. cerevisiae*: (A) outline of experimental procedure as described in Ma *et al.,* 2012. Budding yeast cells were synchronised with α-factor and released in the presence of BrdU for 35 min and 105 min, then chased with thymidine; DNA combing analysis was performed as described.

To be continued

(B) Mean initiation frequencies (blue points with standard deviation) and autocorrelation function *C(r,f)* profiles (blue curve, with standard deviation) with fit (red curve), modeled by either considering two processes or (**C)** a single process. The fitness function was defined as the normalised mean square error (NMSE): $\sum(y - y_{ref})^2/\sum(y - \bar{y}_{ref})^2$. Values close to 0 indicate excellent fits. (**D)** Values from fits to autocorrelation profiles. An F-test was used to test whether the second process significantly improve the fit. The test statistic was calculated as $F = df_2 * (\chi_1 - \chi_2)/(df_1 * \chi_2)$, where df_1 indicates the difference in degrees of freedom between the two models, df_2 the degrees of freedom of the model with two processes, χ_1 and χ_2 the sum of squares of misfits for the two models. Small p-values indicates that the addition of the second process significantly improve the fit. (**E)** Normalized correlation coefficients (ρ1, ρ2) between the molecule's *C(r,f)* and *C1(r,f)* and *C2(r,f)* were calculated. The similarity distance between the molecule and each process was defined as 1- ρ1 for the fast process 1 and 1- ρ2 for the slow process 2 and represented on a two orthogonal axis plot. The red diagonal represents points of equal similarity to the two processes. Points above the diagonal are more similar to the fast process and points below are more similar to the slow process. (**F)** Examples of reconstructed fibers for both processes at one replicated fraction.
